# Thiazolidin-4-Ones as Potential Antimicrobial Agents: Experimental and In Silico Evaluation

**DOI:** 10.3390/molecules27061930

**Published:** 2022-03-16

**Authors:** Christophe Tratrat, Anthi Petrou, Athina Geronikaki, Marija Ivanov, Marina Kostić, Marina Soković, Ioannis S. Vizirianakis, Nikoleta F. Theodoroula, Michelyne Haroun

**Affiliations:** 1 Department of Pharmaceutical Sciences, College of Clinical Pharmacy, King Faisal University, Al-Ahsa 31982, Saudi Arabia; mharoun@kfu.edu.sa; 2 Department of Pharmaceutical Chemistry, School of Pharmacy, Aristotle University of Thessaloniki, 54124 Thessaloniki, Greece; anthi.petrou.thessaloniki1@gmail.com; 3 Mycological Laboratory, Department of Plant Physiology, Institute for Biological Research, Siniša Stankovic-National Institute of Republic of Serbia, University of Belgrade, Bulevar despota Stefana 142, 11000 Belgrade, Serbia; marija.smiljkovic@ibiss.bg.ac.rs (M.I.); marina.kostic@ibiss.bg.ac.rs (M.K.); mris@ibiss.bg.ac.rs (M.S.); 4 Laboratory of Pharmacology, School of Pharmacy, Aristotle University of Thessaloniki, 54124 Thessaloniki, Greece; ivizir@pharm.auth.gr (I.S.V.); theodorn@pharm.auth.gr (N.F.T.); 5 Department of Life and Health Sciences, University of Nicosia, Nicosia CY-1700, Cyprus

**Keywords:** thiazolidine-4-one, antibacterial, antifungal, microdilution method, docking, MurB, CYP51, MRC-5

## Abstract

Herein, we report computational and experimental evaluations of the antimicrobial activity of twenty one 2,3-diaryl-thiazolidin-4-ones. All synthesized compounds exhibited an antibacterial activity against six Gram-positive and Gram-negative bacteria to different extents. Thus, the MIC was in the range of 0.008–0.24 mg/mL, while the MBC was 0.0016–0.48 mg/mL. The most sensitive bacterium was *S*. Typhimurium, whereas *S. aureus* was the most resistant. The best antibacterial activity was observed for compound 5 (MIC at 0.008–0.06 mg/mL). The three most active compounds **5**, **8**, and **15**, as well as compound **6**, which were evaluated against three resistant strains, MRSA, *P. aeruginosa*, and *E. coli*, were more potent against all bacterial strains used than ampicillin. The antifungal activity of some compounds exceeded or were equipotent with those of the reference antifungal agents bifonazole and ketoconazole. The best activity was expressed by compound **5**. All compounds exhibited moderate to good drug-likeness scores ranging from −0.39 to 0.39. The docking studies indicated a probable involvement of *E. coli* Mur B inhibition in the antibacterial action, while CYP51 inhibition is likely responsible for the antifungal activity of the tested compounds. Finally, the assessment of cellular cytotoxicity of the compounds in normal human MRC-5 cells revealed that the compounds were not toxic.

## 1. Introduction

Currently, antimicrobial resistance has become a global problem because, in the contemporary era of travel and trade, resistant organisms rapidly cross the artificial borders through humans or the food chain [1,2]. According to the literature, the cause of antibiotic resistance includes the unreasonable use of antibiotics and lack of new antimicrobial agents with novel mechanisms of action. Only two such antimicrobial agents have been approved by the FDA in the last thirty years: linezolid and daptomycin.

In addition to the well-known genetic mechanisms of AMR, bacteria are able to display some other strategies to resist antimicrobials. The ability to produce biofilms is one of them. Biofilms are implicated in a broad range of infections, making up about 80% [3]. It is responsible for a large variety of those chronic such as periodontitis, endocarditis, urinary and prostate infections. A number of bacteria such as Gram-positive bacteria *Staphylococcus aureus* and *Streptococcus pneumonia* and Gram-negative pathogens such as *E. coli* and *P. aeruginosa* are often responsible for biofilm-associated infections, which are tremendously demanding to treat [4]

Biofilm is a multilayer of highly harmonized microorganisms attached to surfaces in the presence of moisture. Bacterial cells within the biofilm that are highly coordinated create a community that is resistant to the unfavorable outer environment. Such phenotype control also promotes the appearance of antibiotic-resistant genes and the broadcast of genes associated with antibiotic-resistance or genetic mutation [5,6]. Biofilm formation plays a critical role in bacterial infection and antimicrobial resistance, as biofilm-embedded bacteria are more resistant to common antimicrobial agents and host protection systems than bacteria in the planktonic state [7].

Thus, there is an increasing demand for the preparation of new antibacterial agents due to the developing resistance toward conventional antibiotics.

Benzothiazole, a heterocyclic organic compound, and its derivatives have been reported to possess a wide range of biological activities such as antimicrobial [8,9,10,11], anti-inflammatory [12,13], antioxidant [14,15], analgesic [16,17], anticancer [18,19,20], antiviral [21,22,23], anti-HIV [24,25], antidiabetic [26,27], anticonvulsant [28,29], antimalarial [30], and antitubercular [31,32].

**Figure 1:**
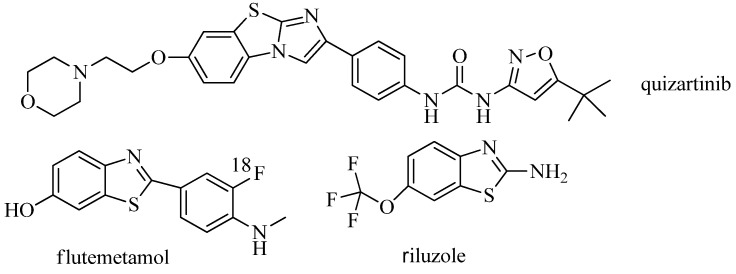
Approved drugs with benzothiazole scaffold.

Furthermore, the benzothiazole scaffold is present in three FDA-approved drugs (Figure 1), which are quizartinib—a receptor tyrosine kinase inhibitor, flutemetamol—a diagnostic tool for Alzheimer’s disease, and riluzole—a drug for the treatment of amyotrophic lateral sclerosis.

**Figure 2:**
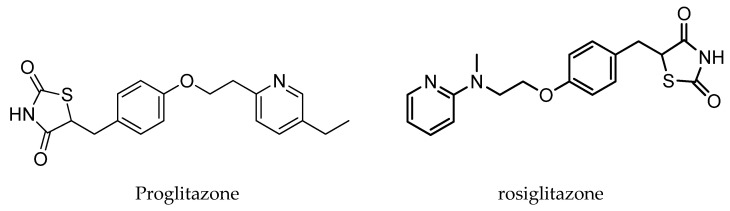
Approved drugs with thiazolidinone scaffold.

On the other hand, the thiazolidinone core has attracted the interest of researchers due to its various degrees of pharmacological and medicinal activities, such as antimicrobial [33,34,35], anti-inflammatory [36,37], anticancer [38,39], antiviral [40,41], antitubercular [38,42], antidiabetic [43], antioxidant [44,45], anticonvulsant [46,47], antimalarial [48], and carbonic anhydrize inhibitors [49,50]. Furthermore, the thiazolidinone moiety is present in the structure of many approved drugs. Thus, proglitazone and rosiglitazone are used to treat type II diabetes (Figure 2).

Prompted by all those mentioned above, as well as based on our previous results [40,41], we herein report the design, the synthesis of new derivatives by the incorporation of two pharmacophores benzothiazole and thiazolidinone moieties in the frame of one molecule, the biological evaluation of synthesized compounds, and the molecular docking studies.

## 2. Results and Discussion

### 2.1. Chemistry

**Scheme 1:**
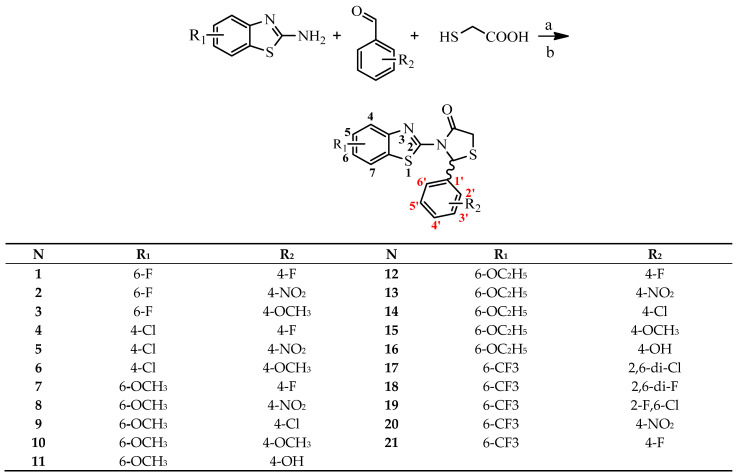
Synthesis of titled compounds.

Synthesis of the compounds was performed by the one-pot method, as described in our previous papers [41] (Scheme 1).

### 2.2. Biological Evaluation

#### 2.2.1. Antibacterial Action

The antimicrobial activity of the synthesized compounds was evaluated using the microdilution method for determining the minimal inhibitory and minimal bactericidal/fungicidal concentrations. The results of antibacterial activity evaluation are shown in Table 1.

All compounds showed good antibacterial activity against the bacteria used, with a MIC range of 0.008–0.24 mg/mL. The order of activity of the compounds tested was **5** > **15** > **8** > **20** > **1** > **6** = **12** > **16** > **18** > **4** > **17** > **10** > **3** > **11** > **13** > **14** > **7** > **2** > **19** > **21** > **9**. The sequence of activity showed that compounds **5**, **15**, and **8** exhibited the best antibacterial activity against all the bacteria used.

The best bacteriostatic and bactericidal activity was exhibited by compound **5** with an MIC range of 0.008–0.06 mg/mL and an MBC of 0.03–0.12 mg/mL, while compound **9** showed the lowest activity among all compounds tested with MIC/MBC values of 0.12–0.48 mg/mL and 0.24–0.96 mg/mL, respectively.

It should be noted that the bacteria generally showed different sensitivities to the test compounds. Thus, the most susceptible bacterium appeared to be the Gram-negative *En. cloacae,* while the Gram-positive *S. aureus* was the most resistant. Compound **5** exhibited excellent activity against *B. cereus* (MIC/MBC at 0.008/0.016 mg/mL), which was 3 and 12.5 times more potent than streptomycin and ampicillin, respectively. On the other hand, this compound showed very good activity also against *M. luteus*, *S.* Typhimurium*, En. cloacae*, *P. aeruginosa*, and *L. monocytogenes* with an MIC of 0.015 mg/mL and MBC of 0.03 mg/mL, again being more potent than streptomycin (1.6–10 fold) and ampicillin (3–13 times). The same good activity was observed for compounds **15** and **16** against *B. cereus*. Compound **8** displayed good activity against all bacteria except *S. aureus* and *S.* Typhimurium with an MIC/MBC of 0.02/0.04 mg/mL. Good activity against *B. cereus* was also observed for compounds **13**, **14**, and **18** (MIC/MBC of 0.03/0.06 mg/mL, while compounds **12**, **15**, and **20** displayed the same good activity as the previous one against all bacteria tested except *S. aureus* (**12**) and *S.* Typhimurium (**12**, **20**). On the other hand, compounds **10** and **16** showed good activity against *En. cloacae*, *P. aeruginosa, L. monocytogenes,* and *E. coli*. It should be mentioned that all these compounds displayed an activity better than streptomycin (MIC range of 0.025–0.1 mg/mL) and ampicillin (MIC range of 0.1–0.3 mg/mL).

Specifically, the MIC range of 0.008–0.32 mg/mL and the MBC range of 0.016–0.64 mg/mL were observed for Gram-positive bacteria, whereas for Gram-negative bacteria, the MIC and MBC ranges were 0.015–0.36 mg/mL and 0.03–0.72 mg/mL, respectively.

The most sensitive bacterium among the Gram-negative bacteria was *En. cloacae*, while *S.* Typhimurium was the most resistant. In terms of Gram-positive bacteria, the most sensitive was found to be *B. cereus*, while the most resistant was *S. aureus.* The study of the structure–action relationship revealed that the presence of a nitro group at the 4-position of the benzene ring and the 4-Cl (**5**) and 6-OMe (**8**) substituents of the benzothiazole ring appeared to favor antibacterial activity, while the combination of 4-NO_2_ in benzene with 6-EtO at the benzothiazole ring was not. On the other hand, 6-OEt (**13**) of the benzene ring in combination with 4-NO_2_ in the benzene ring was negative. In the case of 6-F benzothiazole derivatives, the presence of 4-F in the benzene ring showed good activity, while 4-NO_2_-substituted derivative (**2**) was among the less active compounds. What is worse than that was the influence of the chlorine at the 4-position of the benzene ring (compound **9**) in the case of 6-OMe derivatives resulting in a dramatic decrease in activity, with compound **9** being the less active among all tested compounds. The presence of the 2,3-di-Cl substitution of benzene and 6-CF_3_ substitution (**20**) of the benzothiazole ring appeared to have a positive influence on antibacterial activity.

Regarding 2,6-disubstituted derivatives (compounds **17**–**19**), the 2,6-difluoro (**18**) derivative showed the best effect followed by 2,6-dichloro- (**17**), while the 2-chloro and 6-fluoro (**19**) derivative exhibited the lowest activity.

Thus, taking into account all those mentioned above, it is obvious that the activity of compounds depended not only on the nature of substituents and its position in the benzothiazole ring, but also on the nature and position of substituents in the benzene ring.

Four compounds, three the most active (**5**, **15**, **8**) and **6**, were tested for their activity against three resistant strains, namely methicillin-resistant *S. aureus* (MRSA), *Pseudomonas aeruginosa (P. aeruginosa*), and *Escherichia coli (E. coli*) (Table 2). All compounds appeared to be more potent than ampicillin against all three bacterial species, while streptomycin did not show a bactericidal effect. No one compound was able to stop biofilm formation.

#### 2.2.2. Indifferent Effect in Combination with Streptomycin

Four compounds were evaluated for their interactions with antibiotic streptomycin using the checkboard assay. All of the examined compounds were indifferent from streptomycin (FICI 3, Table 3), suggesting that no synergistic effect could be accomplished with their combining with the examined antibiotic.

#### 2.2.3. Efficient *P. aeruginosa* Bactericidal Effect

**Figure 3:**
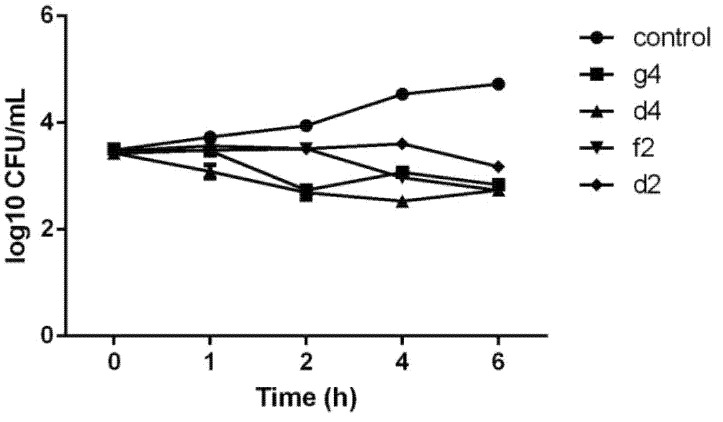
*P. aeruginosa* CFU (log10) after different time intervals of antimicrobial treatment with the MBC of tested compounds.

The application of all four compounds at their MBC induced a reduction in the number of *P. aeruginosa* CFUs. The lowest CFU rate was accomplished after the application of **6** for 4 h. The compounds after 2 h of application already induced a significant reduction in the bacterial growth (Figure 3).

#### 2.2.4. Antifungal Action

The results of antifungal activity are presented in Table 4. As reference compounds, bifonazole and ketoconazole were used.

All compounds showed good antifungal activity against the fungi used, with an MIC range of 0.015–0.24 mg/mL and MFC range of 0.03–0.48 mg/mL. The order of activity of the compounds can be presented as: **5** > **16** > **8** > **6** > **1** > **2** > **9** = **12** = **20** > **15** > **17** > **13** > **3** = **14** > **4** > **10** > **18** > **11** > **21** > **7** > **19**. The best antifungal activity was observed for compound **5** with an MIC of 0.01–0.02 mg/mL and MFC of 0.02–0.04 mg/mL followed by compound **16** with an MIC/MFC of 0.0–0.02/0.02–0.04 mg/mL, while the lowest activity was found for compound **19** with an inhibitory concentration of 0.24–0.48 mg/mL and fungicidal range of action of 0.48–0.96 mg/mL. Compound **5** showed excellent activity against all Aspergillus species with an MIC/MFC of 0.01/0.02 mg/mL, while compounds **8** and **6** exhibited the same good activity against *A. fumigatus, A. ochraceous* (**6**, **8**), and *A. versicolor* (**8**). On the other hand, compound **16** displayed excellent activities against *T. viride* and *P.v.c*, the most resistant fungus, with an MIC and MFC of 0.01 mg/mL and 0.02 mg/mL, respectively, also being very active against all Aspergillus species (MIC/MFC of 0.02/0.04 mg/mL). The same good activity was observed for compound **9** against *A. fumigatus, A. ochraceous, T. viride,* and *P. funiculosum* and **17** against *A. fumigatus, A. ochraceous,* and *A. versicolor*. The reference compounds, bifonazole and ketoconazole, showed fungistatic activity at 0.1–0.2 mg/mL and 0.2–1.0 mg/mL, respectively, while their fungicidal activity was 0.20–0.5 mg/mL and 0.3–1.5 mg/mL, respectively. It should be noted that most of the compounds studied showed a much better activity than the reference compounds. Another positive result of this study was that compounds were active against *A. fumigatus*, as a member of the Aspergillus genus that frequently causes human fungal infections, associated with significant mortality, estimated as a cause of 90% of Aspergillus infections [38], especially in patients with an immunodeficiency.

The study of the structure–action relationship showed that the presence of -nitro substitution at the 4-position of benzene and chloro-substitution at the 4-position of benzothiazole was the most favorable combination for the antifungal activity (compound **5**), as in the case of antibacterial activity, while the replacement of 4-NO_2_ by 4-OMe (**6**) slightly decreased the activity. The replacement of 4-Cl by 6-OEt and 4-OMe by the 4-OH group in benzothiazole and the benzene ring, respectively, led to the second most active compound (**16**), while the combination of 6-OMe and the 4-NO_2_ group resulted in being slightly less active than the previous compound **8**. Replacement of the methoxy group with ethoxy at position 6 of benzothiazole gave compound **13** with dramatically reduced activity.

With respect to the 2,6-substituted derivatives (compounds **17**–**19**), compound **17** (2,6-di-Cl derivative) showed the best activity. Replacement of one F of the benzene ring with Cl had a dramatic effect on activity, with compound **19** exhibiting the lowest activity among all compounds tested.

Thus, it can be concluded that the action is affected by the type and position of the substituent on both the benzene and the benzothiazole rings.

A comparison of the results of the antibacterial and antifungal activities revealed that all the compounds under study did not behave in the same way against bacteria and fungi. Thus, compounds **5**, **8**, and **6** were shown to exhibit equally good antibacterial and antifungal activity, occupying the first positions in the sequence of activity against both bacteria and fungi, while the same was not observed for the rest of compounds. Therefore, according to the results of the present study, it is evident that compound **5** exhibited the best antibacterial and antifungal activity followed by **8** and **6**.

### 2.3. Docking Studies

In order to determine the exact mechanism of the antimicrobial and antifungal activity of the compounds, theoretical binding studies have been demonstrated in various antimicrobial and antifungal targets.

#### 2.3.1. Docking on Antimicrobial Targets

To predict the mechanism of the antibacterial activity of the compounds, theoretical binding studies were performed on the enzymes: *E. coli* MurB (PDB code: 2Q85), *E. coli* DNA GyrB (PDB code: 1KZN), and Thymidyl kinase (PDB code: 4QGG). The results of this study of the compounds showed that the values of free energy of binding for DNA Gyrase (−1.28–−7.15 kcal/mol) and for Thymidylic kinase (−2.26–−4.66 kcal/mol) were higher than those for *E. coli* MurB (−5.73–−12.33 kcal/mol). Therefore, *E. coli* MurB is the most ideal enzyme where the values of free binding energy are in line with biological activity (Table 5).

MurB is an enzyme belonging to the flavoprotein superfamily and plays a role in cell wall biosynthesis in peptidoglycan biosynthesis, which is a complex process involving several steps [51]. Mur B participates in the second stage of the synthesis of peptidoglycan, a key structural component of the bacterial cell wall.

**Figure 4:**
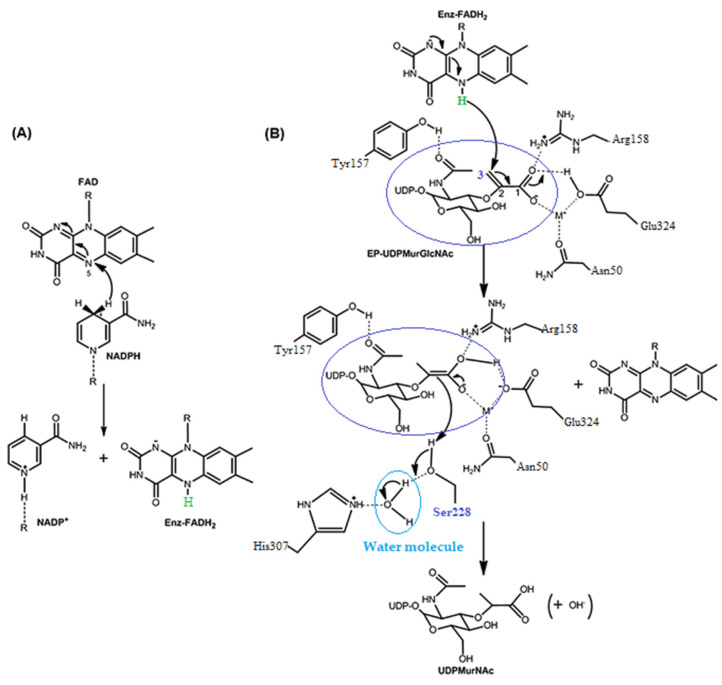
MurB-catalyzed reactions. The first reaction of the transfer of 4-pro-S hydride from NADPH to N-5 of Enz-FAD (**A**) [53] and the transfer of hydride from Enz-FADH2 to C-3 of its enopyrobic portion of EP-UDPGlcNAc (**B**) [54].

The overall reaction catalyzed by MurB comprises two reactions (Figure 4) in which the enzyme-bound FAD serves as the redox intermediate.

The crystal structure of the MurB-EP-UDP-GlcNAc complex showed that the amino acid Ser228 is located at a distance of 3.1 Å from carbon C-2 and in a suitable orientation to serve as a catalytic amino acid and a proton donor to the enolic intermediate formed during the second stage (Figure 4B) [51,52].

According to the results of the docking studies of the compounds to the enzyme MurB (2Q85), it was observed that the most active compound **5** (R-isomer) adopts a configuration within the active center of the enzyme that allows the formation of a hydrogen bond between the residue Ser228 and the C=O group of the thiazolidinedione moiety of the compound (distance: 2.32 Å). As mentioned, the role of the amino acid Ser228 is catalytic to the action of the enzyme [52], so this hydrogen bond plays an important role in the inhibitory activity of the compound.

**Figure 5:**
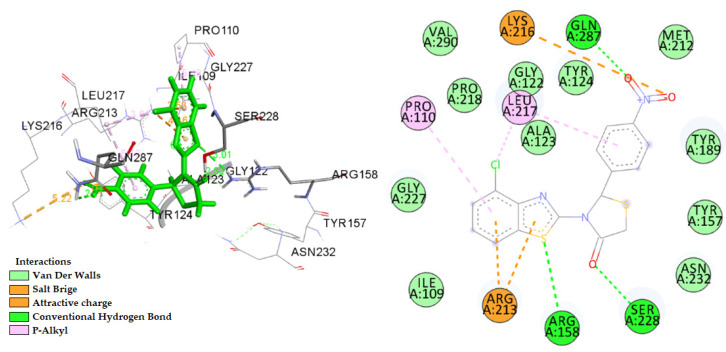
Docking pose of the most active compound **5** (green) in the active site of the *E. coli* MurB enzyme. Dotted green lines represent the hydrogen bonds.

In addition, the formation of two more hydrogen bonds with the residues Gln287 and Arg158 (distance: 2.11 Å and 3.01 Å, respectively) was observed, as well as a number of pi interactions with the residues Lys216, Leu217, Pro110, and Arg213, which contribute to the further stabilization of the enzyme–inhibitor complex and justify the good activity of compound **5** (Figure 5).

**Figure 6:**
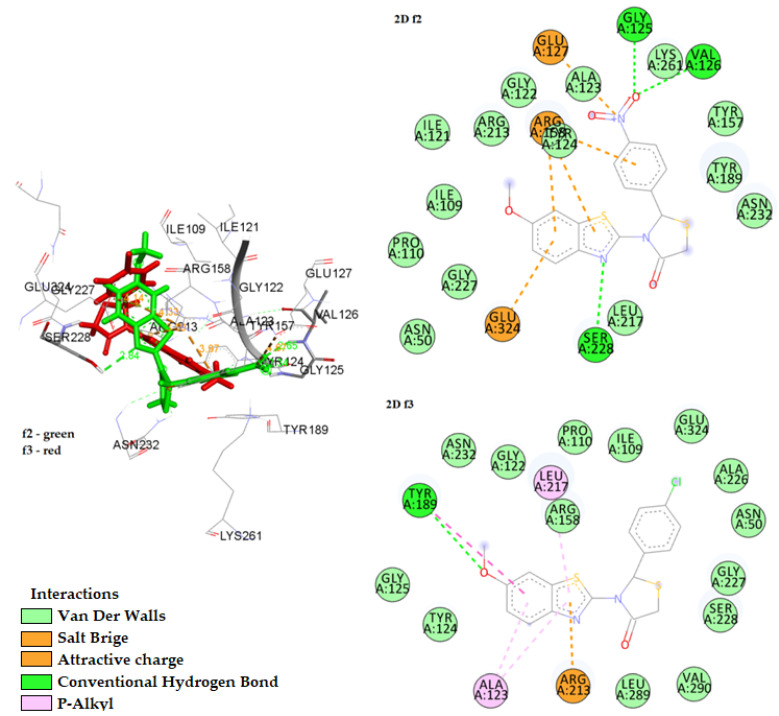
(**Left**) Docking pose of compounds **f2** (green) and **f3** (red) in the active site of the enzyme. (**Right**) 2D diagram of compounds **8** and **9**. The hydrogen bond is represented by a dotted green line.

Hydrogen bond formation with the residue Ser228 was also observed in the case of compound **8** (R-isomer), which is the third in the sequence of activity of the compounds. Replacement of the nitro substitution with chlorine at position 4 of the benzene ring gives compound **9**, which did not exhibit a high inhibitory effect and appeared to take a completely different position at the active site of the enzyme. Finally, only one hydrogen bond was observed and not with the amino acid Ser228 of the enzyme (Figure 6).

As observed from the results of Table 5, the most active compounds showed similar activity against different bacteria. This observation was due to the structural similarity of the active site of MurB in the various bacteria [55].

A docking study of the most active compounds **5**, **8**, and **15** in structures 4JAY, 3TX1, and 1HSK showed that these compounds can form stable complexes with these enzymes, with estimated free energies of binding ranging between −7.13 and −10.18 kcal/mol. The presence of hydrogen bonds, as well as pi–pi interactions with the residues of the active centers of the enzymes, further stabilized the complexes (Table 6, Figure 5 and Figure 6). It should be mentioned that stacking interactions between heterocycles and the aromatic amino acid side chains Phe, Tyr, and Trp are a very attractive topic [56] in the frame of medicinal chemistry owing to the fact that heterocyclic scaffolds prevail between drugs in the market [57,58,59,60]. Boyarskiy et al. [61] gave a detailed analysis of the π–π noncovalent interaction involving an oxadiazole system.

**Figure 7:**
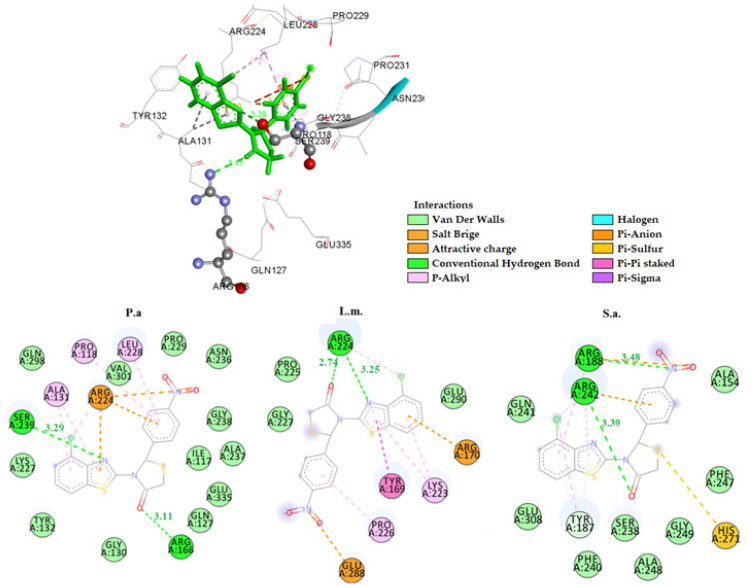
(**Above**) Binding pose of compound **5** (R-isomer) in the active MurB site of *Pseudomonas aeruginos*a (4JAY). (**Bottom**) 2D binding plots of compound **5** to MurB enzymes of *Pseudomonas aeruginosa* (4JAY), *Listeria monocytogenes* (3TX1), and *Staphylococcus aureus* (1HSK), (**left** to **right**).

The docking pose of compound **5** in the MurB enzyme of *Pseudomonas aeruginosa* (4JAY) is shown in Figure 5. Two hydrogen bonds were formed with the amino acids Arg166 and Ser239 (distances 3.11 Å and 3.29 Å, respectively). Pi–pi interactions were also observed between the benzene ring and the residues Leu228 and Pro118, as well as between the benzothiazole and the residues Arg224 and Ala131 (Figure 7).

The binding of **5** to MurB of *Listeria monocytogenes* (3TX1) and *Staphylococcus aureus* (1HSK) is shown in Figure 7. It is worth noting that in the case of the binding of **5** to MurB of *Staphylococcus aureus*, the low free energy of binding was strengthened by the development of a hydrogen bond between the C=O group of the thiazolidinedione moiety and the residue Arg242, which has been considered as an important amino acid involved in the binding of the natural substrate of the enzyme MurB (enolpyruvyl-Uuc-E-c-N-acetyl) [62].

**Figure 8:**
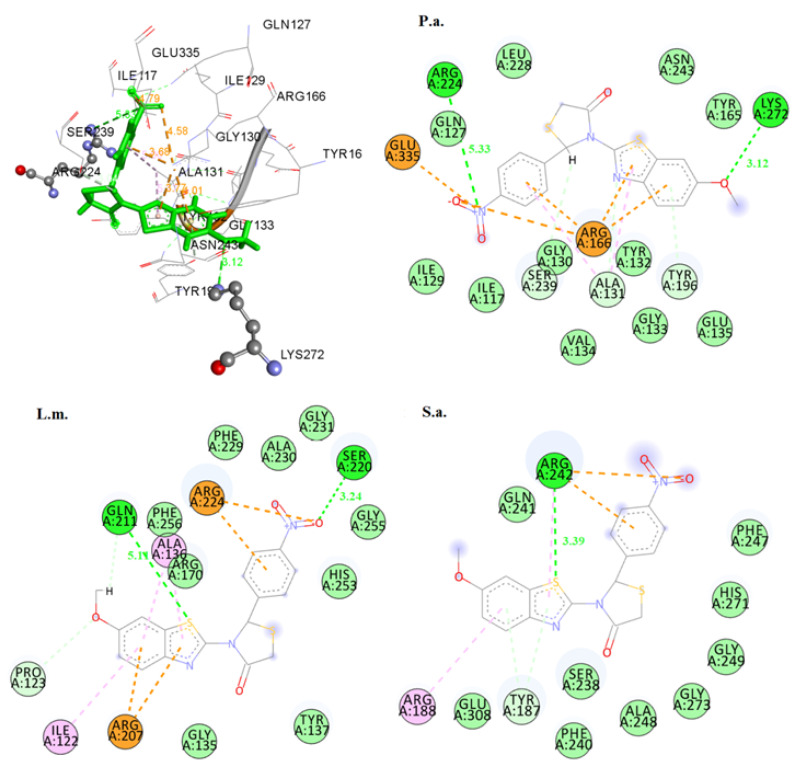
(**Above**) Binding pose of compound **8** (R-isomer) in the active center of MurB *Pseudomonas aeruginosa* (4JAY) and 2D diagram. (**Bottom**) 2D binding plots of compound **8** to the MurB enzymes of *Listeria monocytogenes* (3TX1) and *Staphylococcus aureus* (1HSK), (**left** to **right**).

The second most active compound **8** showed equally good values of free binding energy with the MurB enzymes of the bacteria *Pseudomonas aeruginosa* (4JAY), *Listeria monocytogenes* (3TX1), and *Staphylococcus aureus* (1HSK) (Figure 8). In the case of MurB of *Pseudomonas aeruginosa* binding, the higher free energy of binding of compound **8** than that of **5** is justified by the fact that the distance between the hydrogen bond formed and the residue Arg224 was 5.33 times large enough to stabilize the complex enzyme–inhibitor. However, the plenty of pi–pi and hydrophobic interactions gave a stable enzyme–inhibitor complex with a low free energy of (−9.02 kcal/mol).

In general, the low values of the calculated free energy of binding of the most active compounds **5** and **8** with the MurB enzyme of *E.c., P.a., L.m.,* and *S.a.* showed that inhibition of MurB enzyme is what may explain the antibacterial activity in all bacteria tested.

#### 2.3.2. Docking Studies on the Enzyme Lanosterol 14-α Demethylase of *Candida albicans*

Most antifungal drugs aim to inhibit the biosynthesis of ergosterol, the major component of the cytoplasmic membrane. According to previous research, many thiazolidinone derivatives have been shown to be more likely to act as lanosterol 14α-demethylase inhibitors [63]. Thus, the enzyme 14-α demethylase of *Candida albicans* ERG11 (CYP51), which is available in the database, PDB code: 5V5Z, was selected for the first analysis in order to investigate the possible mechanism of antifungal action of the compounds (Table 7). Dihydrofolate reductase (PDB code: 4HOF) was also used for docking studies [51].

According to docking studies (Table 7), the estimated free energy of binding of the compounds in the enzyme CYP51 ranged between −5.14 kcal/mol for compound **19**, which also showed the lowest in vitro activity, and −13.93 kcal/mol for the most active compound **5**. Interestingly, the calculated free energy of binding followed the same sequence as the antifungal action, with a change in compounds **19** and **21**. This observation strongly supports the suggestion that the inhibition of CYP51 is probably involved in the antifungal activity of the compounds.

**Figure 9:**
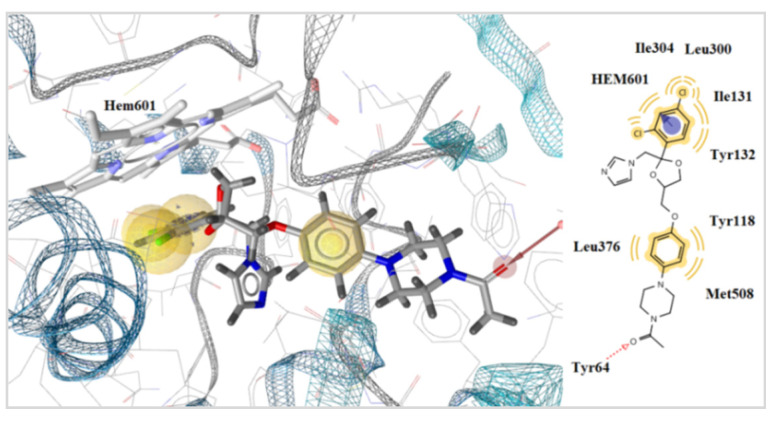
Docking pose of ketoconazole in the active site of *C. albicans* lanosterol 14alpha-demethylase (CYP51ca).

**Figure 10:**
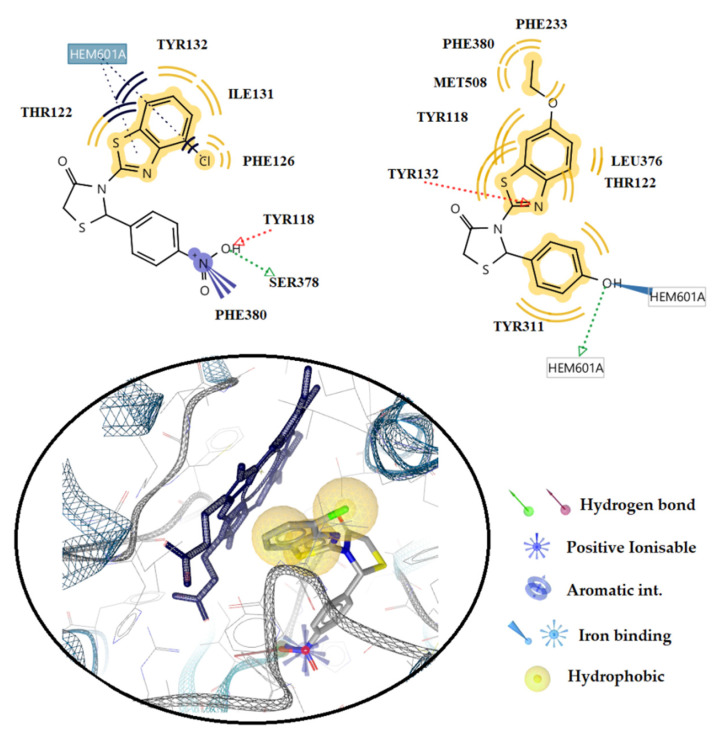
(**Top**) 2D diagrams of compounds **5** (**left**) and **16** (**right**) in the active site of *C. albicans* lanosterol 14alpha-demethylase (CYP51ca). (**Bottom**) Docking pose of compound **5** in the active site of the enzyme lanosterol 14alpha-demethylase of *C. albicans* (CYP51ca). Heme is represented in dark blue.

Docking studies on all compounds studied as well as on the reference drug, ketoconazole, showed that all bind in the same way to CYP51Ca. The most active compound **5** showed two hydrogen bonds between the oxygen atom of the nitro group of the benzene ring and the H atom of the side chains of residues Tyr118 and Ser378 (distance: 2.74 Å and 2.58 Å, respectively). In addition, hydrophobic interactions were also observed between the residues Tyr122, Tyr132, Ile131, and Phe126 and the benzothiazole moiety of compound **5**. The heme group of the protein exhibited positively ionized interactions with the nitrogen atom of the nitro group of compound **5**, as well as hydrophobic interactions with the benzothiazole moiety and the chlorine atom. Hydrophobic interactions with heme were also observed in the reference drug ketoconazole via its benzene moiety and chlorine atoms (Figure 9 and Figure 10). Interactions with the heme molecule of the enzyme occurred in most of the compounds studied; in fact, compound **16** formed a hydrogen bond between the hydrogen atom of the hydroxyl of the benzene ring and the nitrogen of the heme, as well as interaction with iron. This interaction stabilizes the compound–enzyme complex and justifies the higher activity of the compound than ketoconazole (Figure 8, above).

Finally, the calculated free energies of binding in the *Candida albicans* dihydrophylate reductase enzyme were high enough to conclude that this enzyme is not a target of the studied compounds for antifungal activity (Table 7).

### 2.4. In Silico Predictive Studies

Drug-likeness is one of the qualitative ideas employed for predicting drug-like properties. It is designated as an intricate balance of diverse molecular and structural features, which plays a pivotal task in establishing whether the specific drug candidate is alike the known drugs or not. The targeted molecules were appraised for predicting the drug-likeness based on five separate filters, namely Egan [64], Ghose [65], Muegge [66], Veber [67], and Lipinski [68] rules, accompanying bioavailability and drug-likeness scores using the Molsoft software and SwissADME program (http://swissadme.ch, accessed on 20 December 2019) using the ChemAxon’s Marvin JS structure drawing tool.

**Figure 11:**
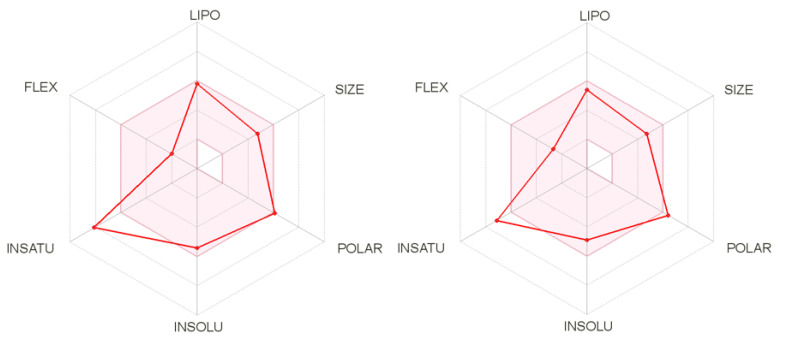
Bioavailability radar of compounds **5** and **8**. The pink area represents the optimal range for each property for oral bioavailability (Lipophilicity (LIPO): XLOGP3 between −0.7 and +5.0, Molecular weight (SIZE): MW between 150 and 500 g/mol, Polarity (POLAR) TPSA between 20 and 130 Å^2^, Solubility (INSOLU): log S not higher than 6, Saturation (INSATU): fraction of carbons in the sp3 hybridization not less than 0.25, and Flexibility (FLEX): no more than 9 rotatable bonds).

The bioavailability and drug-likeness scores of all compounds are given in Table 8. The results showed that none of the compounds violated any rule and their bioavailability score was around 0.55. All compounds exhibited moderate to good drug-likeness scores ranging from −0.38 to 0.42. Moreover, the bioavailability radar of some of the compounds is displayed in Figure 11. Compound **14** appeared to be the best in the in silico predictions with a drug-likeness score of 0.42 without any rule violation.

### 2.5. Cytotoxicity Assessment

#### 2.5.1. Cytotoxicity Assessment of the Synthesized Molecules in the Human Cancer HSC-3 Cell Line

**Figure 12:**
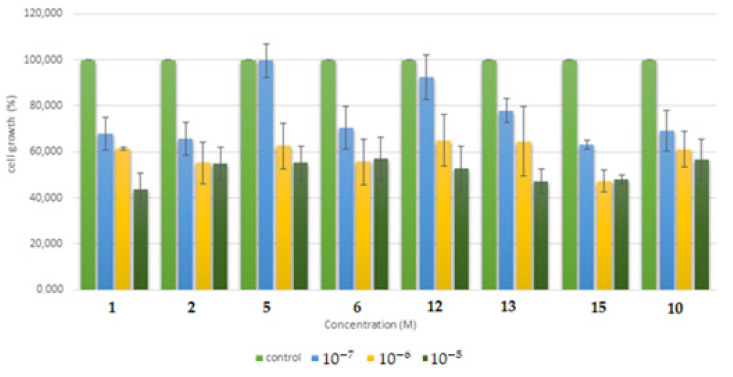
Cell growth kinetics of HSC-3 cells exposed for 48 h at various concentrations in culture to compounds **1**, **2**, **5**, **6**, **12**, **13**, **15**, and **10**.

To assess the cytotoxicity of compounds **1**, **2**, **5**, **6**, **10**, **12**, **13**, and **16** in vitro, the human oral squamous carcinoma HSC-3 cells were exposed for 48 h in culture within the concentration range of 1 × 10^−7^ M–1 × 10^−5^ M. The obtained data are shown in Figure 12. At a concentration of 1 × 10^−5^ M, compound 1 exhibited a cell growth of 44%, following at the order of lower inhibitory capacity by compound **1** (43.7%), **13** (47.2%), **16** (48%), **5** and **2** (52%), **12** (52.8%), **3** (57%), and **10** (57%). Moreover, at a concentration of 1 × 10^−6^ M, the cell growth capacity was 47.2% for compound **16**, 54.9% for **2**, 55.6% for **3**, 61.2% for **10**, 61.5% for **1**, 62.6% for **5**, 64.6% for **13**, and 65% for **12**. In addition, at a concentration 1 × 10^−7^ M, the cell growth capacity for the compounds was: 63% for **16**, 65.8% for **2**, 67.8% for **1**, 69.1% for **10**, 70.5% for **3**, 78% for g2, 92.5% for **12,** and 99.7% for **2**.

As far as the effect on cells is concerned, the compounds showeed very low cytotoxicity capacity in human HSC-3 cells, as, even after 48 h of exposure at the higher tested concentration of 1 × 10^−5^ M, the cell proliferation rate in cultures was around 50% for all the compounds ranging from 43.7% to 57%.

#### 2.5.2. Cytotoxicity Assessment of the Synthesized Molecules in the Human Normal MRC-5 Cell Line

**Figure 13:**
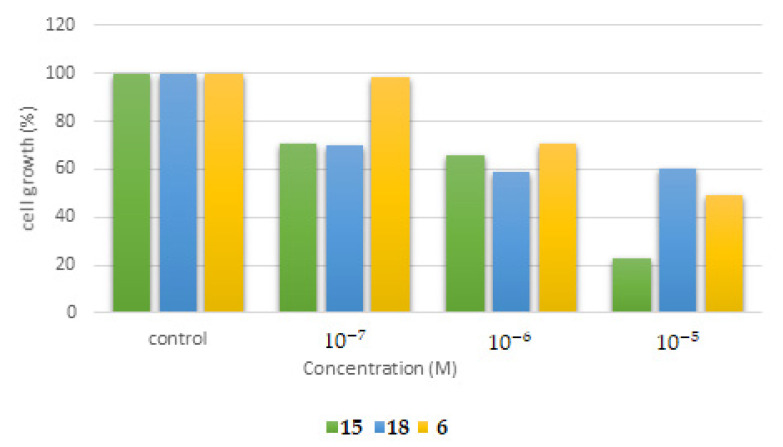
Cell growth kinetics of MRC-5 cells exposed for 48 h at various concentrations in culture to compounds **15**, **18**, and **6**.

Further, to evaluate cytotoxicity, compounds **15**, **18**, and **6** were chosen to be tested in the human normal fetal lung fibroblast MRC-5 cell line within the concentration range of 1 × 10^−7^ M–1 × 10^−5^ M. As shown in Figure 13, after 48 h of exposure in culture, compound **15** exhibited the most prominent inhibition of cell proliferation followed by **6** and then **18**. In particular, upon exposure to 1 × 10^−5^ M, the cell growth for compound **15** was 22.8%, that for compound **6** was 49.4%, and that for compound **18** was 60%. In addition, at a concentration of 1 × 10^−6^ M, the cell proliferation was >59% for all compounds, whereas at a concentration of 1 × 10^−7^ M, only compounds **15** and **18** exhibited limited cytotoxicity, as 70% of cell growth was achieved in culture.

## 3. Materials and Methods

### 3.1. Chemistry

All tested compounds were synthesized and characterized as described in our previous paper [41]. More over ^1^H and ^13^C-NMR of all the compounds are presented in Supplementary Materials.

### 3.2. Biological Evaluation

#### 3.2.1. Antibacterial/Antifungal Action

The following Gram-negative bacteria: *Escherichia coli* (ATCC 35210), *Enterobacter cloacae* (ATCC 35030)*, Salmonella* Typhimurium (ATCC 13311), and *Pseudomonas aeruginosa* (ATCC 27853); Gram-positive bacteria: *Listeria monocytogenes* (NCTC 7973), *Bacillus cereus* (clinical isolate), *Micrococcus luteus* (ATCC 10241), and *Staphylococcus a*ureus (ATCC 6538); and fungi *Aspergillus niger* (ATCC 6275), *Aspergillus fumigatus* (human isolate), *Aspergillus versicolor* (ATCC 11730), *Aspergillus ochraceus* (ATCC 12066), *Penicillium funiculosum* (ATCC 36839), *Trichoderma viride* (IAM 5061), and *Penicillium verrucosum var. cyclopium* (food isolate) were used. The organisms were obtained from the Mycological Laboratory, Department of Plant Physiology, Institute for Biological Research ”Siniša Stankovic” (Belgrade, Serbia).

The antibacterial and antifungal assays were performed as described previously [69,70,71] by determining the minimum inhibitory (MIC) and bactericidal/fungicidal concentrations (MBCs/MFCs) by serial dilution of compounds in the 96-well microdilution plates (Spectar Cacak, Cacak, Serbia). Compounds were dissolved in 30% ethanol solution serially diluted in Tryptic soy broth (TSB) medium (Torlak, Belgrade, Serbia). The bacterial suspension used was adjusted with sterile saline to a concentration of 1.0 × 10^5^ CFU/mL and the bacterial inoculum was added to the plates. The lowest concentration without visible growth (under a binocular microscope) was defined as the one that completely inhibited bacterial growth (MIC). The MBCs were determined by serial sub-cultivation of 10 μL of well sample into microdilution plates containing 100 μL of broth per well and the further incubation for 24 h. The lowest concentration with no visible growth was defined as the MBC. For the antifungal assay, fungal spores were washed from the surface of agar plates with sterile 0.85% saline containing 0.1% Tween 80 (*v*/*v*). The spore suspension was adjusted with sterile saline to a concentration of approximately 1.0 × 10^5^ in a final volume of 100 μL per well. The stock compounds were dissolved in 30% ethanol. The MIC was determined by a serial dilution of compounds in 96-well microtiter plates in malt extract broth (Torlak, Belgrade, Serbia) after which fungal spores were added. The lowest concentrations without visible growth (under a binocular microscope) after incubation at 28 °C for 72 h were defined as MICs. MFCs were determined by serial sub-cultivation of 2 μL of the well’s content and further incubation at 28 °C for 72 h. The lowest concentration with no visible growth was defined as the MFC. Serially diluted solvent (30% ethanol) was used as the negative control.

Resistant strains used in the microdilution assay were isolates of *S. aureus* (strain isolated from cow), *E. coli* (strain isolated from pig), and *P. aeruginosa* (strain isolated from cat) obtained as described in Kartsev et al. [72]. All experiments were performed in duplicate and repeated three times.

#### 3.2.2. Inhibition of Biofilm Formation

This method was performed as described in [73] with some modifications [74]. The percentage of inhibition of biofilm formation was calculated by the formula:

[(A_620_ control − A_620_ sample)/A_620_ control] × 100

#### 3.2.3. Checkboard Assay

A checkboard assay was used for the determination of interactions among the selected compounds and antibiotic, streptomycin. It was carried out with 96-well microplates containing TSB medium for the resistant *P. aeruginosa* strain, and supplemented with examined compounds in concentrations ranging from 1/16 to 4 × MIC as described previously [75] in the checkboard manner. The microplates were incubated for 24 h at 37 °C. The MIC of combinations of examined compounds with streptomycin was determined as for the antimicrobial assay. The fractional inhibitory concentration index (FICI) was calculated by the following equation:

FICI = FIC1^0^*/*MIC1^0^ + FIC2^0^/MIC2^0^

FIC1^0^ and FIC2^0^ are the MICs of the combination of tested compounds and antibiotics, and MIC1^0^ and MIC2^0^ represent the MIC values of individual agents. The following cut-offs: FIC ≤ 0.5 synergistic, >0.5 < 2 additive, ≥ 2 < 4 indifferent, and FIC > 4 antagonistic effects were used for the discussion of obtained results.

#### 3.2.4. Time Kill Assay

The impact of time on the bactericidal effects of selected compounds was evaluated as described in [74] with some modifications. *P. aeruginosa* cells were incubated with the MBC of compounds in a total volume of 100 µL, which was rubbed into Plate Count Agar plates with a sterile spreader after 1, 2, 4, and 6 h of treatment. Plates were incubated at 37 °C, and the number of colonies was counted after 24 h.

### 3.3. Docking Studies

Τhe program AutoDock 4.2^®^ software was used for the docking stimulation. The free energies of binding (ΔG) of *E. coli* DNA GyrB, Thymidylate kinase, *E. coli* primase, *E. coli* MurB, DNA topoIV, and CYP51 of *C*. *albicans* in complex with the inhibitors were generated using this molecular docking program. The X-ray crystal structures data of all the enzymes used were obtained from the Protein Data Bank (PDB ID: 1KZN, AQGG, 1DDE, JV4T, 2Q85, 1S16, and 5V5Z respectively). All procedures were performed according to our previous paper [55].

#### 3.3.1. Docking Studies for Prediction of the Mechanism of Antibacterial Activity

In order to predict the possible mechanism of antibacterial activity of the tested compounds, the enzymes, *E. coli* DNA GyrB, Thymidylate kinase, *E. coli* primase, *E. coli* MurB, and DNA topoIV, were chosen for docking studies. The docking process methodology was first validated by redocking all the co-crystalized original ligands in the active sites of all enzymes with deviation (RMSD) values from 0.86 to 1.63 Å.

#### 3.3.2. Docking Studies for Prediction of the Mechanism of Antifungal Activity

In order to predict the possible mechanism of antifungal activity of the tested compounds, the enzymes CYP51 14α-lanosterol demethylase and dihydrofolate reductase were used. The X-ray crystal structures 5V5Z and 4HOF, respectively, for each enzyme were obtained for the Protein Data Bank. The docking box was centered on the heme molecule, at the active center of the CYP51 14α-lanosterol demethylase enzyme, both with a target box of 50 × 50 × 50 Å. All selected X-ray crystal structures were in complex with inhibitors. Docking of these inhibitors to their enzyme structures was performed for verification of the method with RMSD values of 0.85 and 1.36 Å for CYP51 14α-lanosterol demethylase and dihydrofolate reductase, respectively. Furthermore, the reference drug ketoconazole was docked into the active site of the 5V5Z structure.

### 3.4. In Silico Predictive Studies

Drug-likeness is one of the qualitative ideas employed for predicting drug-like properties. It is designated as an intricate balance of diverse molecular and structural features, which plays a pivotal task in establishing whether the specific drug candidate is similar to the known drugs or not. The targeted molecules were appraised for predicting the drug-likeness based on 5 separate filters, namely Egan [64], Ghose [65], Muegge [66], Veber [67], and Lipinski [68] rules, accompanying bioavailability and drug-likeness scores using the Molsoft software and SwissADME program (http://swissadme.ch, accessed on 20 December 2019) using the ChemAxon’s Marvin JS structure drawing tool.

### 3.5. Assessment of Cytotoxicity

The normal human lung fibroblast MRC-5 and the human oral squamous carcinoma HSC-3 cells cell lines are stored and used in our laboratory in a routine manner (Dr. I.s. Vizirianakis, Laboratory of Pharmacology, School of Pharmacy, Aristotle University of Thessaloniki, Greece) (passage < 40). Cells were grown in culture (37 °C, humidified atmosphere containing 5% *v*/*v* CO_2_) in DMEM medium supplemented with 10% *v*/*v* FBS and 1% PS penicillin-streptomycin. The compounds tested were dissolved in DMSO and stored at 4 °C. For the assessment of cytotoxicity, the cells were seeded in a 96-well plate at an initial concentration of 5 × 10^4^ cells/mL and allowed to attach for at least 3 h before the addition of the compounds at concentrations of 1 × 10^−5^ M (10 μΜ), 1 × 10^−6^ M (1 μΜ), and 1 × 10^−7^ M (0.1 μΜ). Note that the concentration of DMSO in culture was ≤0.2% *v*/*v*, in which no detectable effect on cell proliferation was observed [41]. To assess the cytotoxicity of each compound, the cells were allowed to grow for an additional 48 h before their number was estimated in culture using the Neubauer counting chamber under an optical microscope. Cell growth in each treated culture was expressed as percentage compared to that seen for the untreated control cells. Moreover, the number of dead cells was also measured using the Trypan-blue method, as previously described [76,77]. Statistical *t*-test analysis was performed via the use of the GraphPad Prism 6.0 program.

## 4. Conclusions

Twenty-one 3-(4/6-substituted -benzo[d]thiazol-2-yl)-2-4(substituted/disubstituted phenyl) thiazolidin-4-one compounds previously designed and synthesized were evaluated in silico and experimentally against the panel of Gram-positive and Gram-negative bacteria, and fungi.

All compounds showed good antibacterial activity, most of them being more potent against almost all strains compared to ampicillin and some of them compared to streptomycin. Thus, compound **5** exhibited excellent activity against *B. cereus*, which were 3 and 12.5 times more potent than streptomycin and ampicillin, respectively, and also against *M. luteus*, *S.* Typhimurium, *En. cloacae*, *P. aeruginosa*, and *L. monocytogenes*, which were 1.6–10-fold, and ampicillin 3–13-fold, more potent than streptomycin and ampicillin, respectively. The most sensitive bacterial strain appeared to be *B. cereus*, while *S. aureus* was the most resistant. The evaluation of the four compound’s activity against three resistant strains MRSA, *E. coli*, and *P. aeruginosa* revealed that all excised the activity of ampicillin against all three bacterial species, while streptomycin did not show a bactericidal effect, but no one compound was able to stop biofilm formation.

It should be mentioned that all four examined compounds were indifferent with streptomycin, but their application at their MBC induced a reduction in the number of *P. aeruginosa* CFUs with compound **6** showing the lowest CFU.

As far as antifungal activity is concerned, all compounds were equipotent or more active than both reference drugs against all fungi, especially against *T. viride* compounds, which were 9 to 33 times more potent than bifonazole.

Docking analysis against different targets (DNA Gyrase, Thymidylate kinase, and *E. coli* MurB) revealed a probable involvement of MurB inhibition in the antibacterial mechanism of compounds tested. On the other hand, the prediction by docking the mechanism of antifungal activity against 14α-lanosterol demethylase (CYP51) and tetrahydrofolate reductase of *Candida albicans* showed a probable implication of CYP51 reductase at the antifungal activity of the compounds.

The results of prediction of the bioavailability and drug-likeness showed that none of the compounds violated any rule and their bioavailability score was around 0.55, while drug-likeness scores ranged from −0.38 to 0.42.

Finally, the evaluation of cytotoxicity of compounds **15**, **18**, and **6** against normal human MRC-5 cell lines showed that only **15** and **18** exhibited limited cytotoxicity, as 70% of cell growth was achieved in culture at a concentration of 1 × 10^−7^ M. As far as the effect on cells is concerned, the compounds showed very low cytotoxicity capacity in human HSC-3 cells, as even after 48 h of exposure, at the higher tested concentration of 1 × 10^−5^ M, the cell proliferation rate in cultures was around 50% for all the compounds, ranging from 43.7% to 57%.

## Figures and Tables

**Table 1:** Antibacterial activity of compounds (MIC/MBC in mg/mL).

Compounds		*B.c.*	*S.a.*	*M.l.*	*L.m.*	*P.a.*	*En.cl.*	*E.c.*	*S.T.*
**1**	**MIC**	0.045	0.045	0.045	0.045	0.045	0.045	0.045	0.09
	**MBC**	0.09	0.09	0.09	0.09	0.09	0.09	0.09	0.18
**2**	**MIC**	0.06	0.24	0.06	0.24	0.24	0.03	0.03	0.24
	**MBC**	0.12	0.48	0.12	0.48	0.48	0.06	0.06	0.48
**3**	**MIC**	0.06	0.24	0.06	0.06	0.06	0.06	0.06	0.24
	**MBC**	0.12	0.48	0.12	0.12	0.12	0.12	0.12	0.48
**4**	**MIC**	0.03	0.12	0.06	0.12	0.06	0.06	0.06	0.12
	**MBC**	0.06	0.24	0.12	0.24	0.12	0.12	0.12	0.24
**5**	**MIC**	0.008	0.06	0.015	0.015	0.015	0.015	0.015	0.015
	**MBC**	0.016	0.12	0.03	0.03	0.03	0.03	0.03	0.03
**6**	**MIC**	0.03	0.06	0.06	0.06	0.06	0.03	0.06	0.06
	**MBC**	0.06	0.12	0.12	0.12	0.12	0.06	0.12	0.12
**7**	**MIC**	0.08	0.32	0.08	0.08	0.08	0.08	0.08	0.32
	**MBC**	0.16	0.64	0.16	0.16	0.16	0.16	0.16	0.64
**8**	**MIC**	0.02	0.08	0.02	0.02	0.02	0.02	0.02	0.08
	**MBC**	0.04	0.16	0.04	0.04	0.04	0.04	0.04	0.16
**9**	**MIC**	0.48	0.48	0.12	0.48	0.12	0.12	0.12	0.24
	**MBC**	0.96	0.96	0.24	0.96	0.24	0.24	0.24	0.48
**10**	**MIC**	0.03	0.24	0.06	0.03	0.06	0.03	0.03	0.24
	**MBC**	0.06	0.48	0.12	0.06	0.12	0.06	0.06	0.48
**11**	**MIC**	0.24	0.24	0.06	0.06	0.06	0.06	0.06	0.12
	**MBC**	0.48	0.48	0.12	0.12	0.12	0.12	0.12	0.24
**12**	**MIC**	0.03	0.12	0.03	0.03	0.03	0.03	0.03	0.12
	**MBC**	0.06	0.24	0.06	0.06	0.06	0.06	0.06	0.24
**13**	**MIC**	0.03	0.24	0.06	0.24	0.06	0.06	0.06	0.24
	**MBC**	0.06	0.48	0.12	0.48	0.12	0.12	0.12	0.48
**14**	**MIC**	0.03	0.24	0.24	0.06	0.06	0.03	0.03	0.24
	**MBC**	0.06	0.48	0.48	0.12	0.12	0.06	0.06	0.48
**15**	**MIC**	0.015	0.03	0.03	0.03	0.03	0.03	0.03	0.03
	**MBC**	0.03	0.06	0.06	0.06	0.06	0.06	0.06	0.06
**16**	**MIC**	0.015	0.12	0.12	0.03	0.03	0.03	0.03	0.12
	**MBC**	0.03	0.24	0.24	0.06	0.06	0.06	0.06	0.24
**17**	**MIC**	0.08	0.08	0.08	0.08	0.08	0.08	0.08	0.08
	**MBC**	0.16	0.16	0.16	0.16	0.16	0.16	0.16	0.16
**18**	**MIC**	0.03	0.06	0.06	0.03	0.06	0.03	0.03	0.24
	**MBC**	0.06	0.12	0.12	0.06	0.12	0.06	0.06	0.48
**19**	**MIC**	0.1	0.4	0.2	0.1	0.1	0.1	0.1	0.4
	**MBC**	0.2	0.8	0.4	0.2	0.2	0.2	0.2	0.8
**20**	**MIC**	0.03	0.03	0.03	0.03	0.03	0.03	0.03	0.12
	**MBC**	0.06	0.06	0.06	0.06	0.06	0.06	0.06	0.24
**21**	**MIC**	0.09	0.36	0.18	0.18	0.18	0.18	0.18	0.36
	**MBC**	0.18	0.72	0.36	0.36	0.36	0.36	0.36	0.72
**Streptomycin**	**MIC**	0.025	0.1	0.05	0.15	0.1	0.025	0.1	0.1
	**MBC**	0.05	0.2	0.1	0.3	0.2	0.05	0.2	0.2
**Ampicillin**	**MIC**	0.1	0.1	0.1	0.15	0.3	0.1	0.15	0.1
	**MBC**	0.15	0.15	0.15	0.3	0.5	0.15	0.2	0.2

*B.c.—B. cereus, M.l.—M. luteus, S.a.—S. aureus, L.m.—L. monocytogenes, En.c.—En. cloacae, P.a.—P. aeruginosa, S.t.—S.* Typhimurium, *E.c.—E. coli*.

**Table 2:** Antibacterial activity of selected compounds toward resistant bacterial strains; results are in mg/mL; MIC—minimal inhibitory concentrations; MBC—minimal bactericidal concentration.

Compounds		*MRSA*	*P.a.*	*E.c.*
**5**	**MIC**	0.09	0.18	0.09
	**MBC**	0.18	0.36	0.18
**6**	**MIC**	0.18	0.09	0.09
	**MBC**	0.36	0.18	0.18
**8**	**MIC**	0.36	0.18	0.09
	**MBC**	0.72	0.36	0.18
**15**	**MIC**	0.09	0.09	0.09
	**MBC**	0.18	0.18	0.18
**Streptomycin**	**MIC**	0.1	0.05	0.1
	**MBC**	/	0.1	0.2
**Ampicillin**	**MIC**	/	0.2	0.2
	**MBC**	/	/	/

*MRSA*—Methicillin-resistant *Staphylococcus aureus*, *E.c.*—*E. coli*, *P.a.*—*P. aeruginosa*.

**Table 3:** FICI indexes of combinations of selected compounds with streptomycin.

Compound	FICI
**5**	3
**6**	3
**8**	3
**15**	3

**Table 4:** Antifungal activity of titled compounds (MIC/MFC in mg/mL).

Compounds		*A.f.*	*A.n.*	*A.o.*	*A.v.*	*T.v.*	*P.f.*	*P.v.c.*	*P.o.*
**1**	**MIC**	0.03	0.06	0.03	0.03	0.06	0.03	0.06	0.03
	**MFC**	0.06	0.12	0.06	0.06	0.12	0.06	0.12	0.06
**2**	**MIC**	0.03	0.015	0.03	0.03	0.06	0.06	0.06	0.06
	**MFC**	0.06	0.03	0.06	0.06	0.12	0.12	0.12	0.12
**3**	**MIC**	0.06	0.06	0.06	0.06	0.06	0.06	0.12	0.12
	**MFC**	0.12	0.12	0.12	0.12	0.12	0.12	0.24	0.24
**4**	**MIC**	0.06	0.06	0.06	0.06	0.12	0.12	0.06	0.12
	**MFC**	0.12	0.12	0.12	0.12	0.24	0.24	0.12	0.24
**5**	**MIC**	0.01	0.01	0.01	0.01	0.02	0.02	0.02	0.02
	**MFC**	0.02	0.02	0.02	0.02	0.04	0.04	0.04	0.04
**6**	**MIC**	0.015	0.03	0.015	0.03	0.03	0.03	0.03	0.03
	**MFC**	0.03	0.06	0.03	0.06	0.06	0.06	0.06	0.06
**7**	**MIC**	0.15	0.15	0.15	0.15	0.3	0.3	0.3	0.15
	**MFC**	0.3	0.3	0.3	0.3	0.6	0.6	0.6	0.3
**8**	**MIC**	0.01	0.02	0.01	0.01	0.01	0.01	0.08	0.02
	**MFC**	0.02	0.04	0.02	0.02	0.02	0.02	0.16	0.04
**9**	**MIC**	0.02	0.08	0.04	0.02	0.02	0.02	0.08	0.08
	**MFC**	0.04	0.16	0.08	0.04	0.04	0.04	0.16	0.16
**10**	**MIC**	0.06	0.12	0.06	0.06	0.12	0.06	0.12	0.06
	**MFC**	0.12	0.24	0.12	0.12	0.24	0.12	0.24	0.12
**11**	**MIC**	0.03	0.06	0.06	0.06	0.12	0.12	0.12	0.12
	**MFC**	0.06	0.12	0.12	0.12	0.24	0.24	0.24	0.24
**12**	**MIC**	0.06	0.06	0.06	0.06	0.03	0.03	0.03	0.03
	**MFC**	0.12	0.12	0.12	0.12	0.06	0.06	0.06	0.06
**13**	**MIC**	0.06	0.06	0.03	0.06	0.03	0.06	0.12	0.06
	**MFC**	0.12	0.12	0.06	0.12	0.06	0.12	0.24	0.12
**14**	**MIC**	0.06	0.06	0.06	0.06	0.12	0.06	0.12	0.06
	**MFC**	0.12	0.12	0.12	0.12	0.24	0.12	0.24	0.12
**15**	**MIC**	0.03	0.03	0.03	0.06	0.06	0.03	0.12	0.03
	**MFC**	0.06	0.06	0.06	0.12	0.12	0.06	0.24	0.06
**16**	**MIC**	0.02	0.02	0.02	0.02	0.01	0.01	0.02	0.02
	**MFC**	0.04	0.04	0.04	0.04	0.02	0.02	0.04	0.04
**17**	**MIC**	0.02	0.08	0.02	0.02	0.08	0.04	0.08	0.04
	**MFC**	0.04	0.16	0.04	0.04	0.16	0.08	0.16	0.08
**18**	**MIC**	0.04	0.08	0.04	0.08	0.16	0.08	0.16	0.08
	**MFC**	0.08	0.16	0.08	0.16	0.32	0.16	0.32	0.16
**19**	**MIC**	0.24	0.24	0.24	0.24	0.48	0.48	0.48	0.48
	**MFC**	0.48	0.48	0.48	0.48	0.96	0.96	0.96	0.96
**20**	**MIC**	0.03	0.03	0.03	0.03	0.06	0.06	0.06	0.06
	**MFC**	0.06	0.06	0.06	0.06	0.12	0.12	0.12	0.12
**21**	**MIC**	0.09	0.09	0.09	0.09	0.09	0.18	0.18	0.18
	**MFC**	0.18	0.18	0.18	0.18	0.18	0.36	0.36	0.36
**Bifonazole**	**MIC**	0.15	0.15	0.15	0.1	0.15	0.2	0.1	0.2
	**MFC**	0.2	0.2	0.2	0.2	0.2	0.25	0.2	0.25
**Ketoconazole**	**MIC**	0.2	0.2	0.15	0.2	1	0.2	0.2	1
	**MFC**	0.5	0.5	0.2	0.5	1.5	0.5	0.3	1.5

*A.f.*—*Aspergillus fumigatus* (human isolate), *A.n.*—*Aspergillus niger* (ATCC 6275), *P.f.*—*Penicillium funiculosum* (ATCC 36839), *P.v.c.*—*Penicillium versicolor var. cyclopium, T.v.*—*Trichoderma viride* (IAM 5061), *A.o.*—*Aspergillus ochraceus* (ATCC 12066), *A.v.*—*Aspergillus versicolor* (ATCC 11730), *P.o.*—*Penicillium ochrochloron* (ATCC 9112).

**Table 5:** Results of docking studies on antibacterial targets.

N.	Est. Binding Energy (kcal/mol)				I-H *E. coli* MurB	Residues *E. coli* MurB
	Gyrase 1KZN (R-isomer)	Thymidylate Kinase 4QGG (R-isomer)	*E. coli* MurB 2Q85 (S-isomer)	*E. coli* MurB 2Q85 (R-isomer)		
**1**	−5.81	−3.11	−7.52	−8.25	2	Gly122, Ser228
**2**	−1.79	−2.51	−5.26	−6.15	1	Ser228
**3**	−4.36	-	−6.14	−7.11	2	Gly122, Ser228
**4**	−4.22	-	−7.06	−7.68	2	Arg158
**5**	−5.26	−2.55	−8.66	−10.21	3	Arg158, Ser228, Gln287
**6**	−4.19	−1.25	−6.98	−8.27	2	Arg158, Ser228
**7**	−1.46	-	−5.24	−6.11	1	Ser228
**8**	−5.87	−3.11	−7.83	−9.15	3	Gly125, Val126, Ser228
**9**	−1.96	−1.11	−4.28	−5.17	1	Tyr189
**10**	−4.09	-	−6.52	−7.70	2	Gly122, Ser228
**11**	−3.32	−1.22	−5.49	−6.55	2	Gly122, Ser228
**12**	−4.79	−2.16	−7.26	−8.22	2	Arg158, Ser228
**13**	−2.71	−1.27	−5.18	−6.88	2	Ser228, Arg213
**14**	−1.25	-	−3.28	−6.62	1	Ser228
**15**	−4.27	−2.51	−8.80	−10.08	3	Arg158, Ser228, Asn232
**16**	−3.34	−1.28	−6.29	−8.07	2	Ser228, Ala226
**17**	−2.47	−1.63	−6.33	−7.52	2	Arg158, Asn232
**18**	−3.18	-	−6.17	−7.79	2	Arg158, Tyr189
**19**	−2.87	-	−4.11	−5.16	1	Arg158
**20**	−1.27	-	−3.03	−5.33	1	Asn232
**21**	−2.10	−1.25	−4.57	−5.74	1	

**Table 6:** Results of docking studies of compounds **5**, **8,** and **15** (R-isomers) to the enzymes MurB *Pseudomonas aeruginosa, P.a.,* (4JAY); *Listeria monocytogenes, L.m.,* (3TX1); and *Staphylococcus aureus, S.a*., (1HSK).

N.	Bacteria (Structure)	Est. Binding Energy (kcal/mol)	I-H	Residues	Pi-Interactions
	*P.a.* (4JAY)	−10.18	2	Arg166, Ser239	Ala131, Pro118, Leu228, Arg224
**5**	*L.m.* (3TX1)	−10.03	2	Arg224	Tyr169, Lys223, Arg224, Pro226, Glu228
	*S.a.* (1HSK)	−10.95	2	Arg188, Arg242	Arg188, Tyr187, Arg242, His271
	*P.a.* (4JAY)	−9.02	2	Arg224, Lys272	Ala131, Arg166, Ser239, Tyr196, Glu335
**8**	*L.m.* (3TX1)	−8.77	2	Gln211, Ser220	Ile122, Ala136, Arg207, Arg224
	*S.a.* (1HSK)	−7.13	1	Arg242	Arg188, Tyr187, Arg242
	*P.a.* (4JAY)	−9.11	2	Gly164, Lys242	Arg166, Leu245, Asp268, Arg270
**15**	*L.m*. (3TX1)	−8.23	2	Arg170, Ser220	Met132, Ala134, Arg170, Glu290
	*S.a.* (1HSK)	−10.14	3	Ser238, Lys250, His271	Ala155, Tyr155, Arg188, Phe247, Lys250, His271

**Table 7:** Results of docking study on the enzymes dihydrophylate reductase (DHFR, PDB ID: 4HOF) and 14α-demethylase of lanosterol (CYP51, PDB ID: 5V5Z) of *Candida albicans*.

	Est. Binding Energy				
N.	Dihydrofolate Reductase 4HOF	CYP51 *C. albicans* 5V5Z	I-H	Residues	Interactions with Heme HEM601
**1**	−3.61	−7.39	1	Tyr132	Hydrophobic
**2**	−5.19	−10.17	1	Ser378	Hydrophobic
**3**	−3.11	−8.58	1	Tyr64	Hydrophobic
**4**	−4.15	−7.58	1	Tyr132	Hydrophobic
**5**	−6.28	−13.93	2	Tyr118, Ser378	p. ionizable, Hydrophobic
**6**	−4.27	−11.05	1	Tyr118	Hydrophobic
**7**	−1.25	−7.22	1	Tyr118	Hydrophobic
**8**	−5.36	−11.13	1	Tyr132	Hydrophobic
**9**	-	−8.72	1	Tyr64	Hydrophobic
**10**	−2.69	−8.21	1	Tyr118	Hydrophobic
**11**	−3.55	−9.06	1	Hem601	H-bond, Hydrophobic
**12**	−5.15	−9.75	1	Tyr132	Hydrophobic, aromatic
**13**	−5.22	−9.22	1	Ser378	Hydrophobic
**14**	−4.16	−9.15	1	Tyr132	Hydrophobic
**15**	−4.28	−9.23	1	Tyr118	Hydrophobic
**16**	−6.12	−1.98	2	Tyr132, Hem601	H-bond, Hydrophobic
**17**	−2.88	−10.48	1	Tyr132	Hydrophobic, Aromatic
**18**	−1.24	−8.6	1	Tyr118	Hydrophobic
**19**	−1.87	−5.14	-	-	Hydrophobic
**20**	−2.14	−6.17	1	Tyr118	Hydrophobic
**21**	−1.34	−5.82	-	-	Hydrophobic
**Ketoconazole**	−6.75	−8.23	1	Tyr64	Hydrophobic, Aromatic

**Table 8:** Drug-likeness predictions and physicochemical-pharmacokinetic/ADME properties of tested compounds.

No.	MW	Number of HBA ^a^	Number of HBD ^b^	Log *P*_o/w_ (iLOGP) ^c^	Log S ^d^	TPSA ^e^	Lipinski, Ghose, Veber, Egan, and Muegge Violations	Bioavailability Score	Drug-Likeness Model Score
**1**	348.39	4	0	3.09	Moderately soluble	86.74	0	0.55	−0.18
**2**	375.40	5	0	2.28	Moderately soluble	132.56	0	0.55	−0.22
**3**	360.43	4	0	3.19	Moderately soluble	95.97	0	0.55	0.09
**4**	364.84	3	0	3.10	Moderately soluble	86.74	0	0.55	0.38
**5**	391.85	4	0	2.02	Moderately soluble	132.56	0	0.55	0.03
**6**	376.88	3	0	3.23	Poorly soluble	103.23	0	0.55	0.34
**7**	360.43	4	0	3.02	Moderately soluble	95.97	0	0.55	0.28
**8**	387.43	5	0	2.49	Moderately soluble	141.79	0	0.55	−0.17
**9**	376.88	3	0	3.10	Poorly soluble	95.97	0	0.55	0.39
**10**	372.46	4	0	3.45	Moderately soluble	105.20	0	0.55	−0.06
**11**	358.43	4	1	2.56	Moderately soluble	116.30	0	0.55	0.13
**12**	374.45	4	0	3.25	Poorly soluble	95.97	0	0.55	0.30
**13**	401.46	5	0	4.32	Moderately soluble	141.79	0	0.55	−0.17
**14**	390.19	3	0	3.41	Moderately soluble	95.97	0	0.55	0.42
**15**	403.50	5	1	3.27	Moderately soluble	125.43	0	0.55	0.03
**16**	372.46	4	1	2.84	Moderately soluble	116.20	0	0.55	0.14
**17**	449.30	5	0	3.19	Moderately soluble	86.74	1	0.55	−0.31
**18**	416.39	7	0	3.03	Moderately soluble	86.74	0	0.55	−0.38
**19**	432.84	6	0	3.13	Moderately soluble	86.74	0	0.55	−0.32
**20**	425.40	7	0	2.44	Moderately soluble	132.56	0	0.55	−0.40
**21**	398.40	6	0	3.18	Moderately soluble	86.74	0	0.55	−0.00

**^a^** number of hydrogen bond acceptors; **^b^** number of hydrogen bond donors; **^c^** lipophilicity; **^d^** water solubility (SILICOS-IT (S = Soluble)); **^e^** topological polar surface area (Å^2^).

## Data Availability

Not applicable.

## Supplementary Materials

The following supporting information can be downloaded at: https://www.mdpi.com/article/10.3390/molecules27061930/s1, ^1^H-NMR and ^13^C-NMR of compounds **1**–**21**.

## Sample Availability

Samples of the compounds are not available from the authors.
