# Supplementary material for: Thiazolidin-4-Ones as Potential Antimicrobial Agents: Experimental and In Silico Evaluation
Source: Molecules. 2022 Mar 16;27(6):1930. doi: 10.3390/molecules27061930 (PMC8954104; doi:10.3390/molecules27061930)

Article

Thiazolidin-4-ones as potential antimicrobial agents: Experimental and in silico evaluation

Christophe Tratrat [1]*, Anthi Petrou, [2] Athina Geronikaki, *[2] Marija Ivanov [3], Marina Sokovic [3] Ioannis S. Vizirianakis [4,5] Nikoleta F. Theodoroula [4] and Michelyne Haroun [1]

[1] Department of Pharmaceutical Sciences, College of Clinical Pharmacy, King Faisal University, Al-Ahsa 31982, Saudi Arabia; mharoun@kfu.edu.sa (M.H.);
[2] Department of Pharmaceutical Chemistry, School of Pharmacy, Aristotle University of Thessaloniki, 54124 Thessaloniki, Greece; geronik@pharm.,auth.gr; anthi.petrou.thessaloniki1@gmail.com
[3] Mycological Laboratory, Department of Plant Physiology, Institute for Biological Research, Siniša, Stankovic-National Institute of Republic of Serbia, University of Belgrade, Bulevar Despota Stefana 142,11000 Belgrade, Serbia; marija.smiljkovic@ibiss.bg.ac.rs (M.I.); marina.kostic@ibiss.bg.ac.rs (M.K.); mris@ibiss.bg.ac.rs (M.D.S.)
[4] Laboratory of Pharmacology, School of Pharmacy, Aristotle University of Thessaloniki, GR-54124 Thessaloniki, Greece; theodorn@pharm.auth.gr (N.F.T.); ivizir@pharm.auth.gr (I.S.V.)
[5] Department of Life and Health Sciences, University of Nicosia, CY-1700 Nicosia, Cyprus.
* Correspondence: geronik@pharm.auth.gr; Tel.:+302301997616

General procedure for the synthesis of thiazolidinones by conventional method

The appropriate heteroaromatic amine (1.0 mmol) and substituted benzaldehyde (1.2 mmol) were stirred in dry toluene under reflux followed by addition of mercatoacetic acid (2.0 mmol). The reaction mixture was refluxed for 18–32 hours and then concentrated to dryness under reduce pressure. The residue was diluteted in ethyl acetate and the organic layer was washed with 5% aq citric acid, water and 5% aq sodium hydrogen carbonate. The organic layer was dried over sodium sulfate and concentrated under reduced pressure. All the synthesized compounds were characterized by TLC, ^{1}H-NMR and ^{13}C-NMR.

^{1}H-NMR and ^{13}C-NMR of compounds

Compound 1

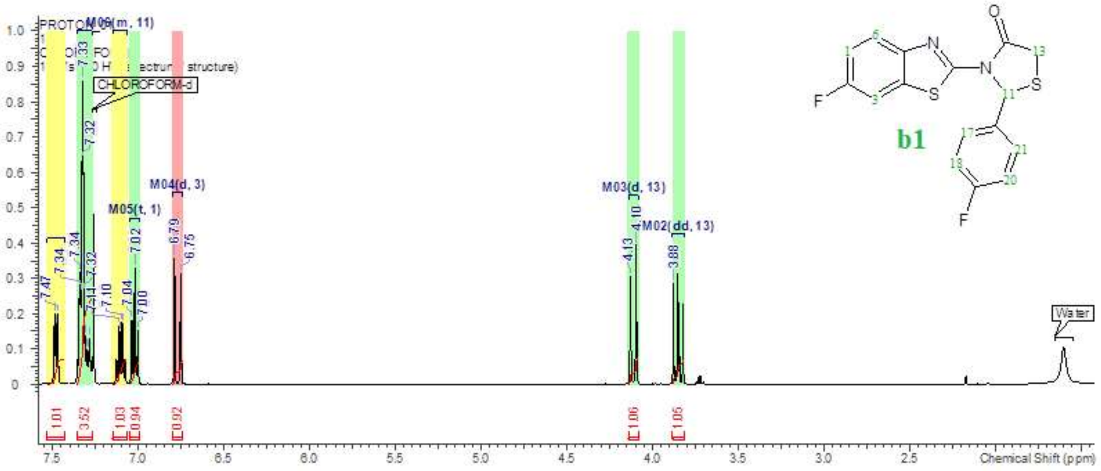

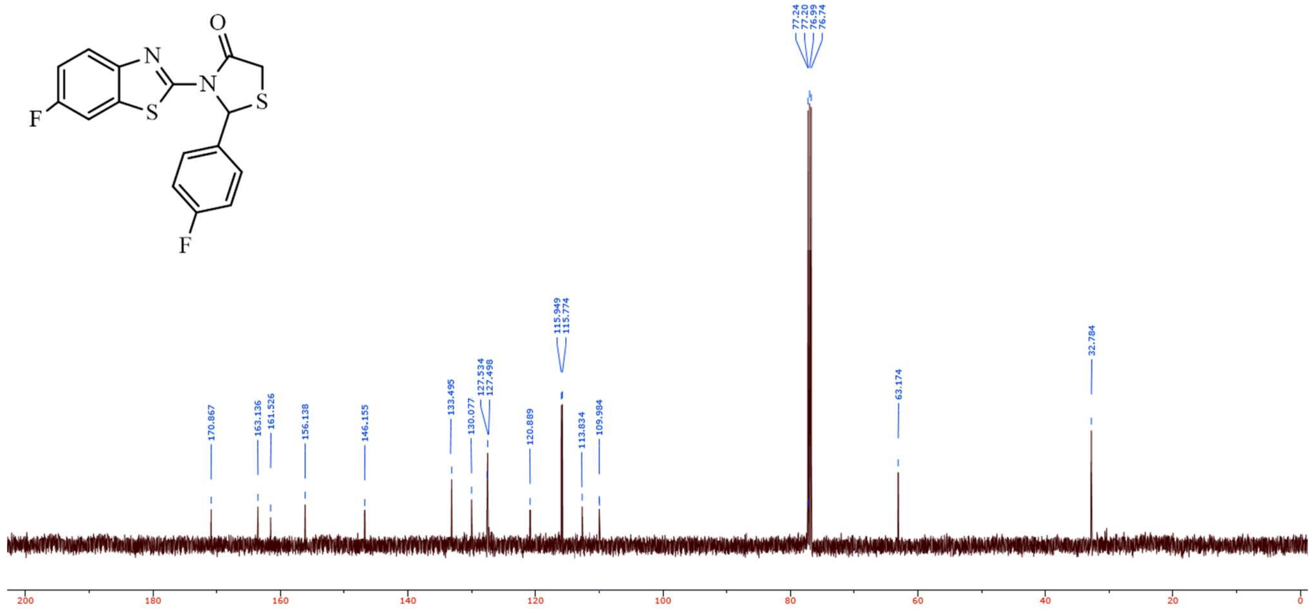

Compound 2

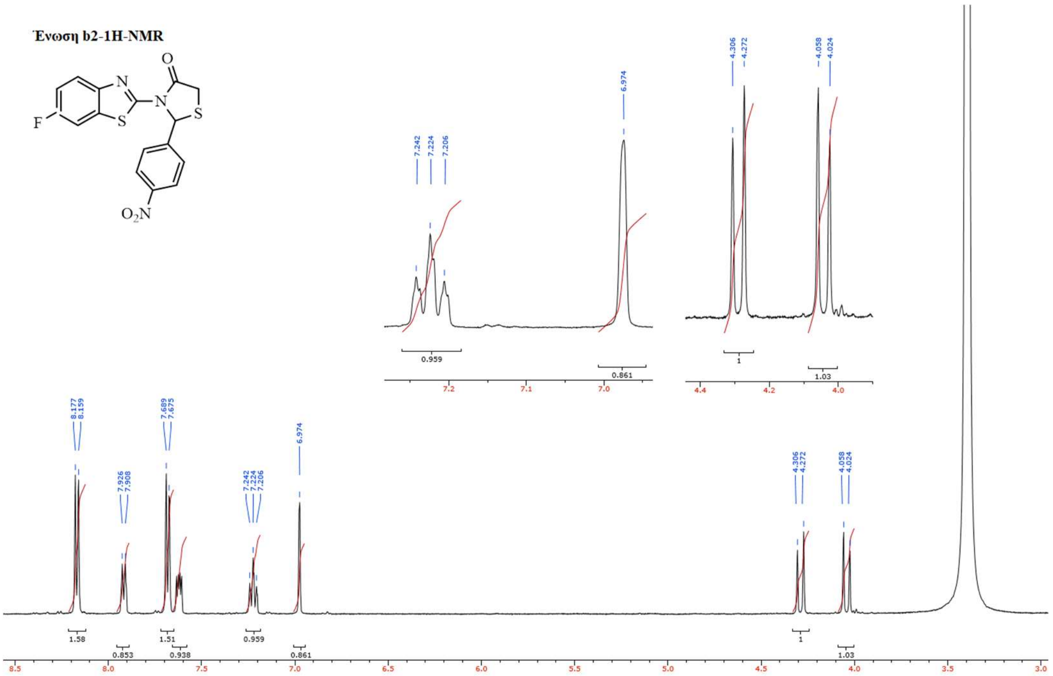

Ένωση b2-1H-NMR
F
N
S
O
S
O2N
8.177
8.159
7.926
7.908
7.689
7.675
7.242
7.224
7.206
6.974
4.306
4.272
4.058
4.024
1.58
0.853
1.51
0.938
0.959
0.861
1
1.03

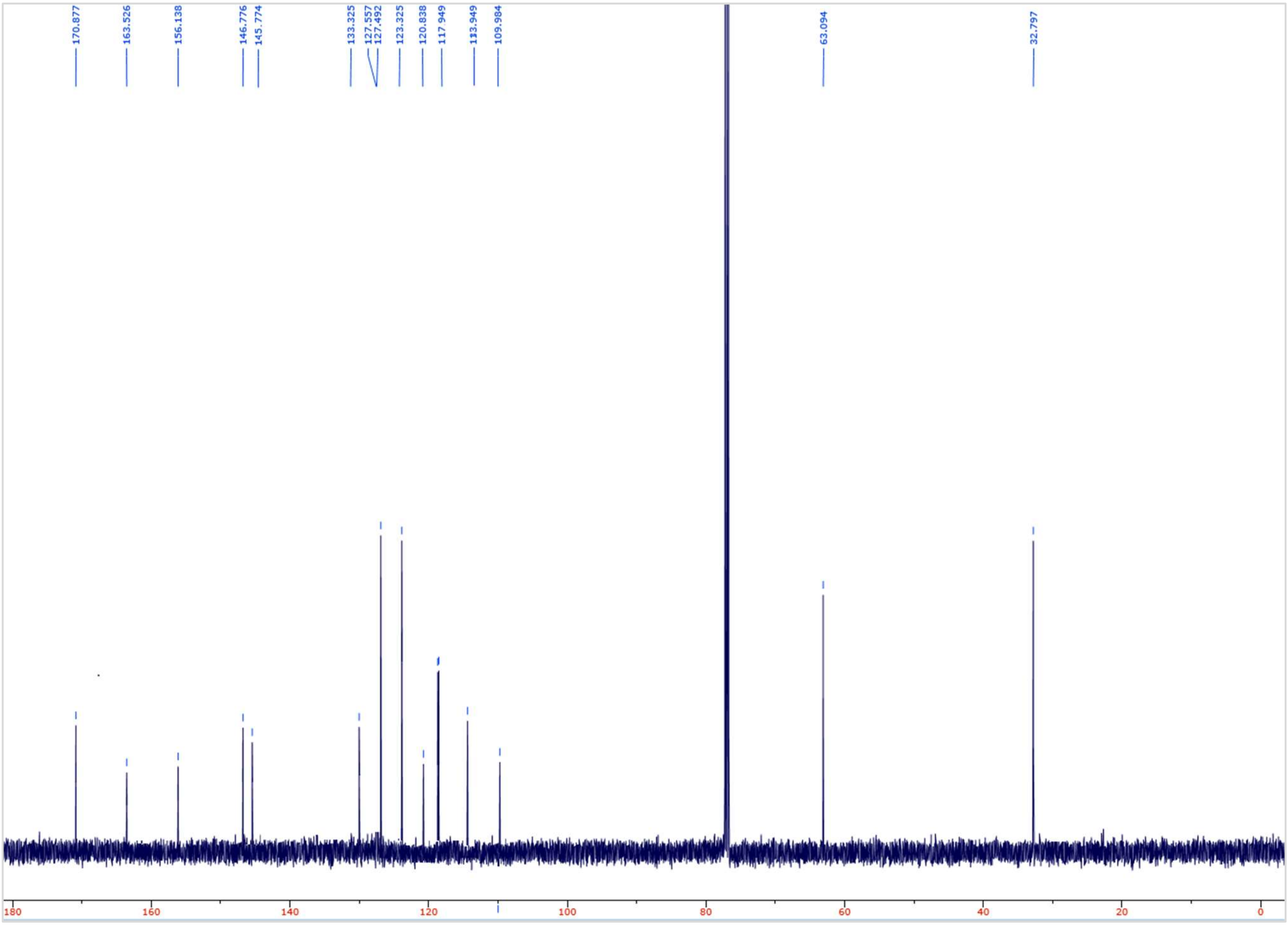

170.877
163.526
156.138
146.776
145.774
133.325
127.557
127.492
123.325
120.838
117.949
113.949
109.984
63.094
32.797

Compound 3

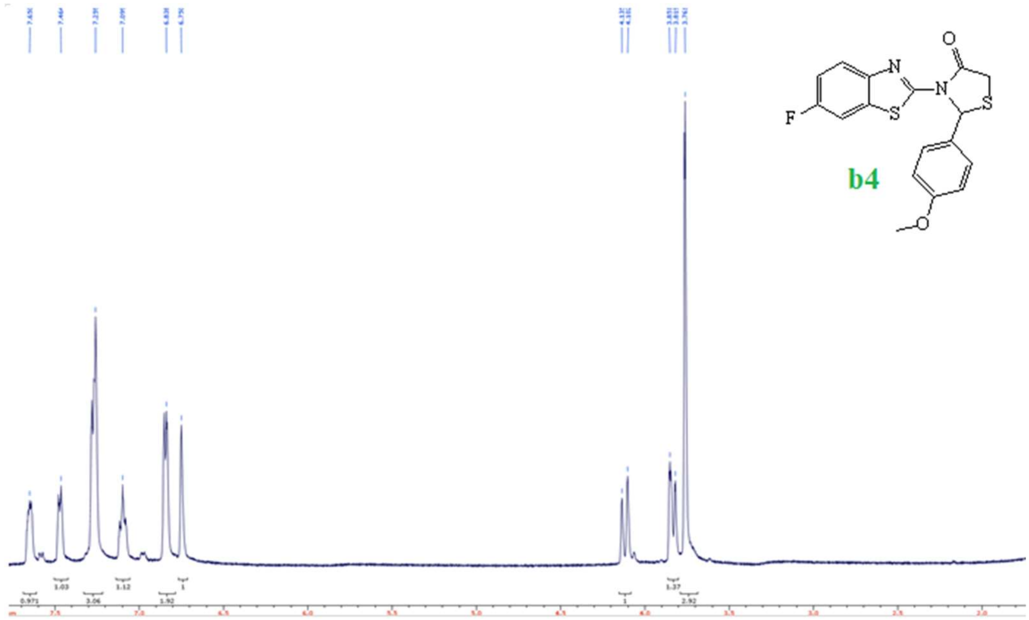
F
N
S
N
O
S
O
b4

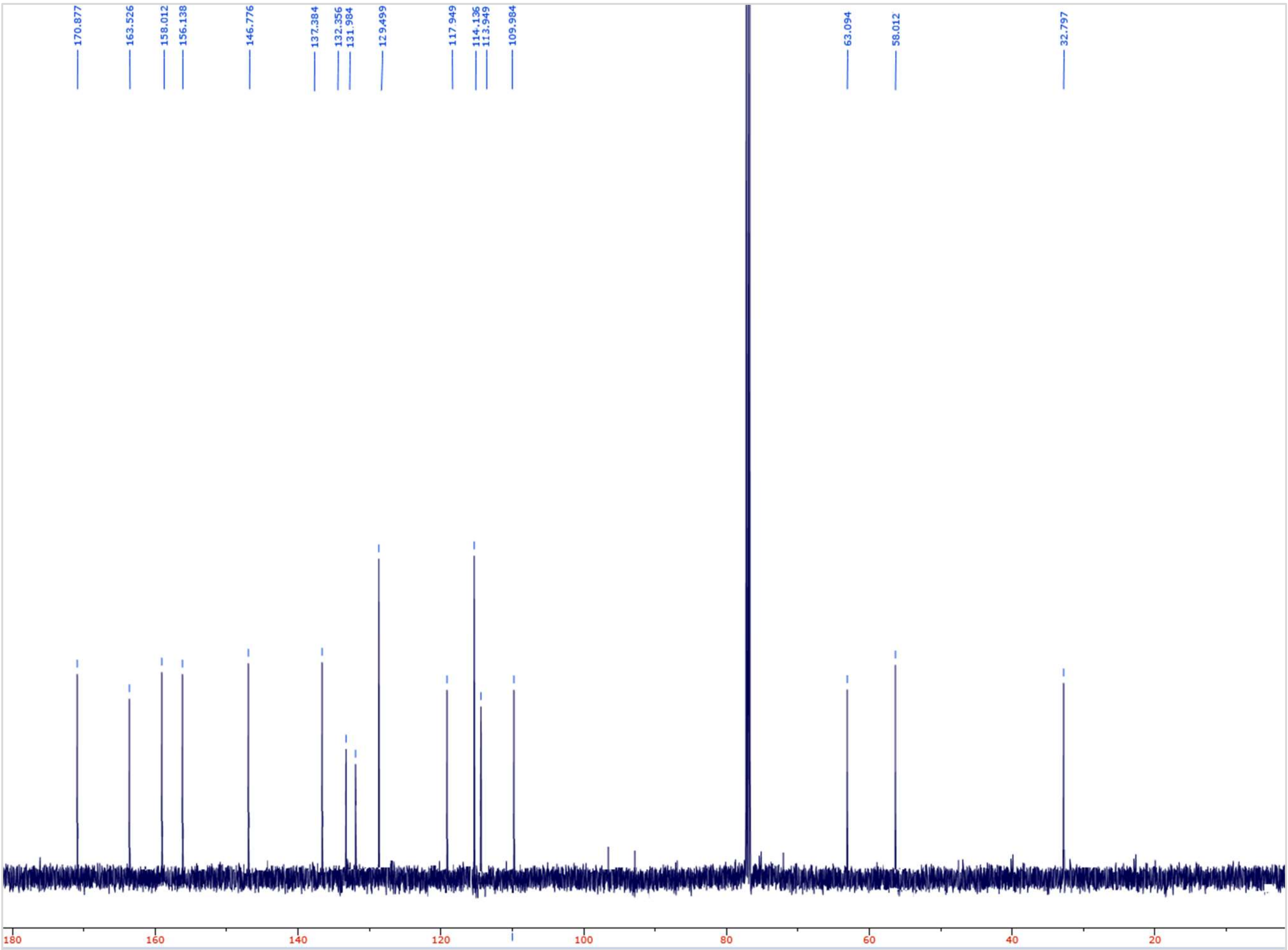
170.877
163.526
158.012
156.138
146.776
137.384
132.356
131.984
129.499
117.949
114.136
113.949
109.984
63.094
58.012
32.797
180
160
140
120
100
80
60
40
20

Compound 4

Ένωση d1 1H-NMR

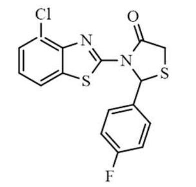

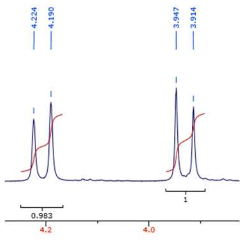

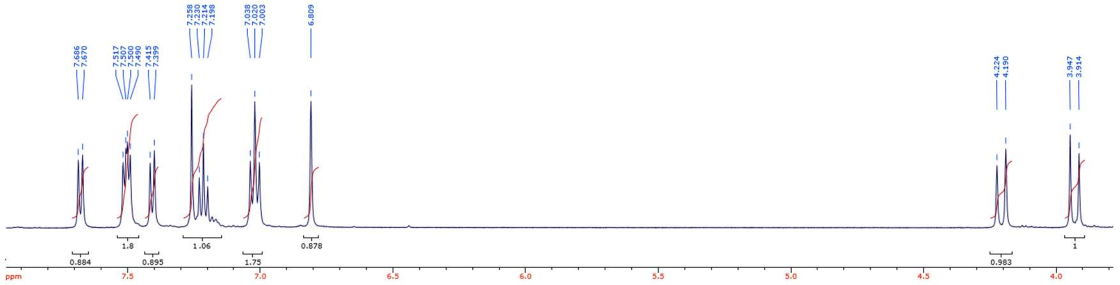

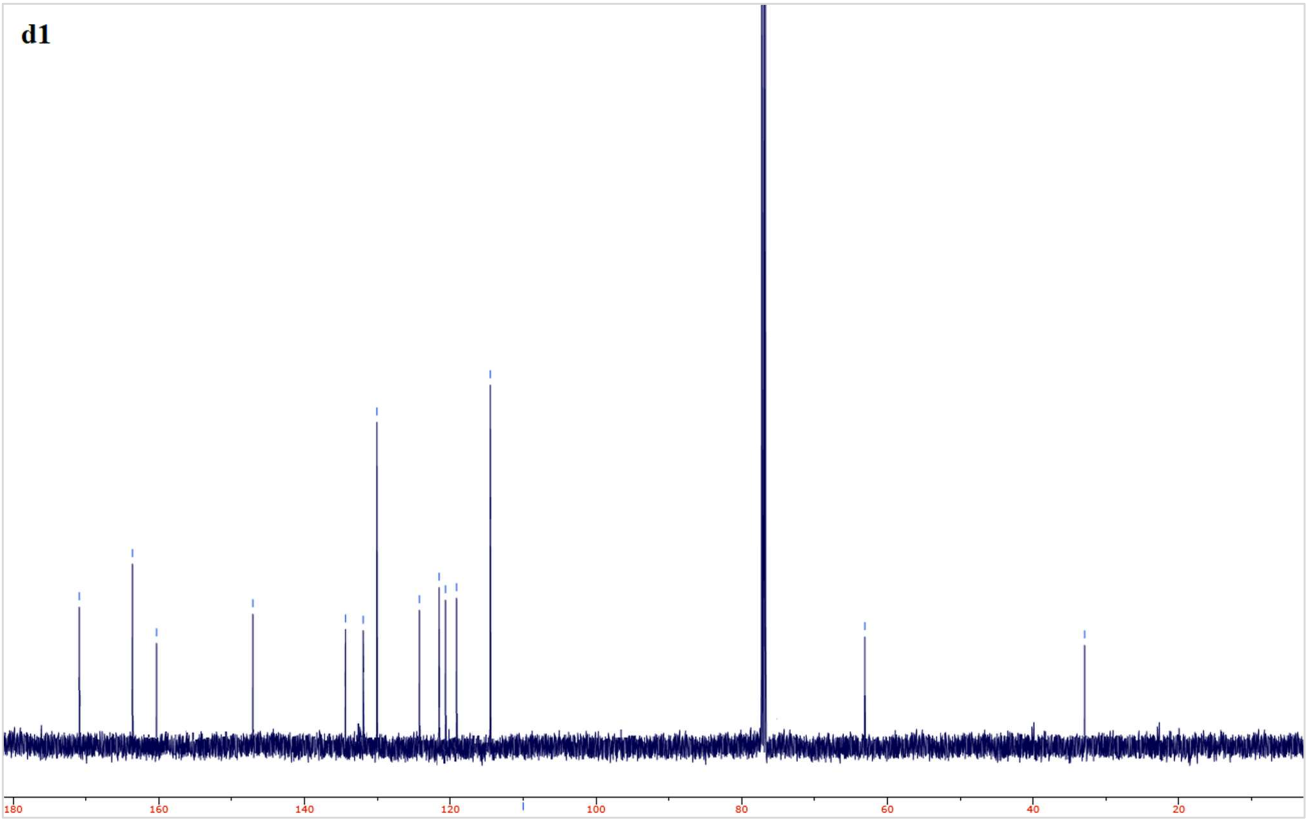

Compound 5

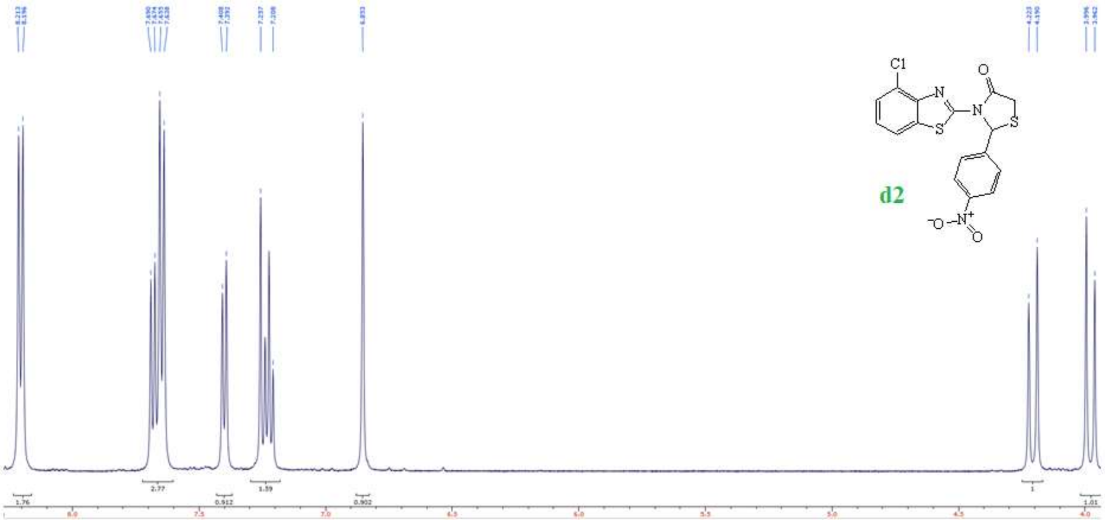

Cl
N
S
O
d2
1.76
2.77
0.912
1.59
0.902
1
1.01
8.0
7.5
7.0
6.5
6.0
5.5
5.0
4.5
4.0

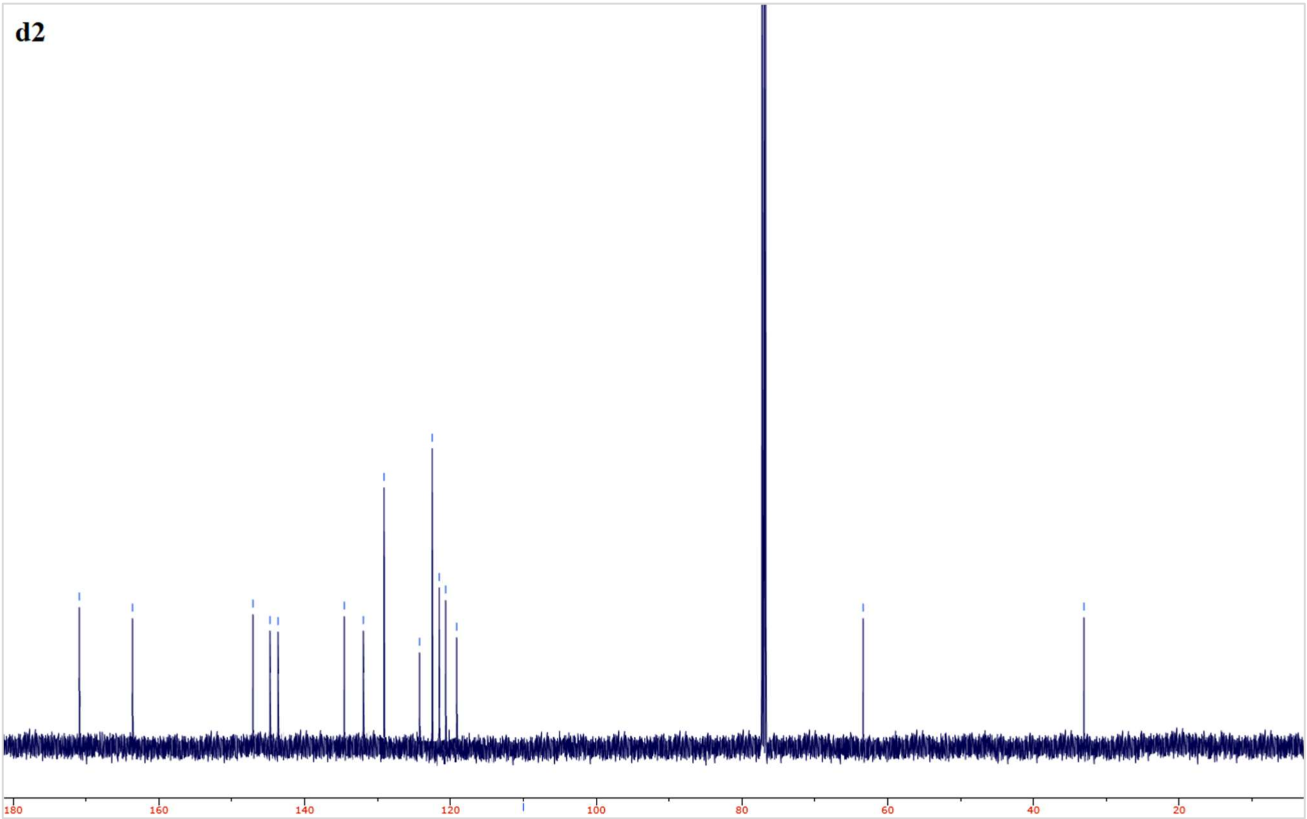

d2
180
160
140
120
100
80
60
40
20

Compound 6

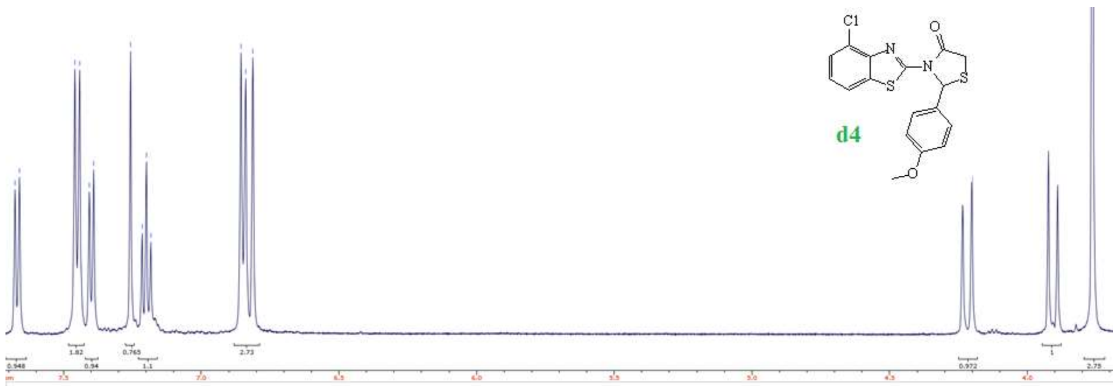
Cl
N
N
O
S
S
O
d4
0.948
1.82
0.94
0.765
1.1
2.73
0.972
1
2.75

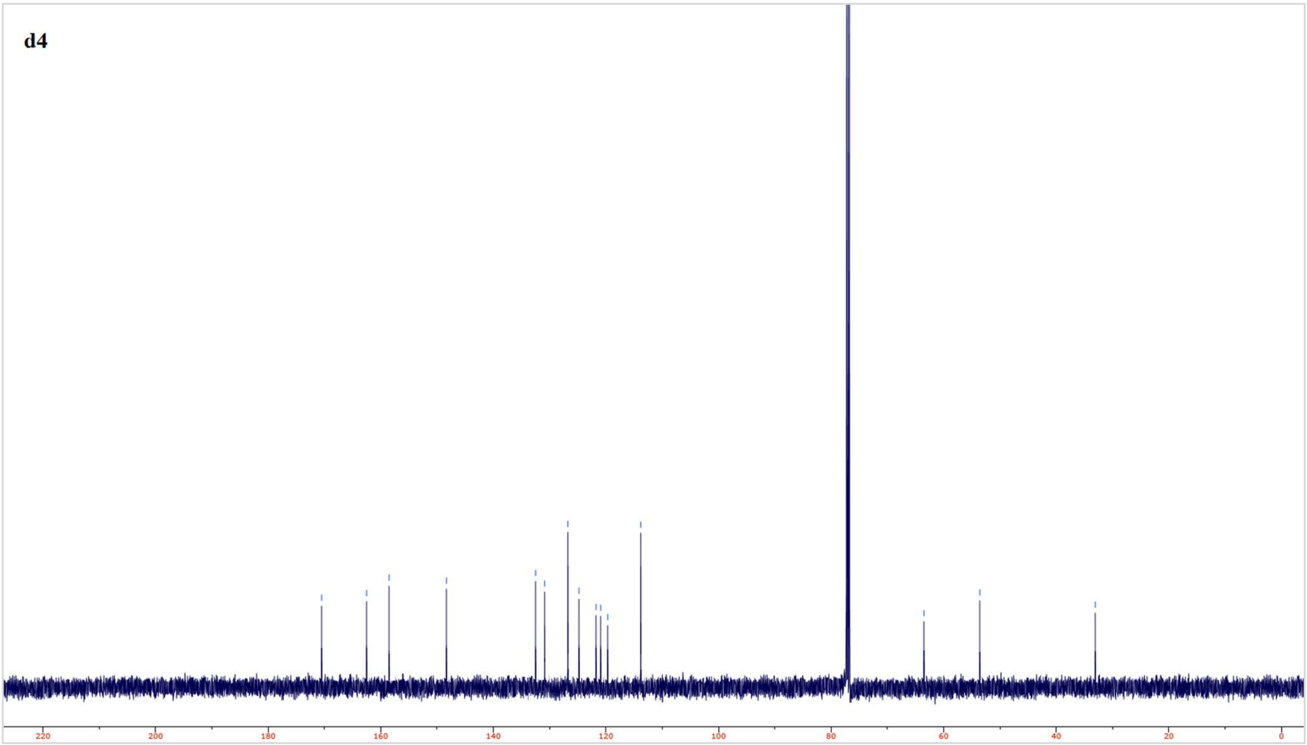
d4
220
200
180
160
140
120
100
80
60
40
20
0

Compound 7

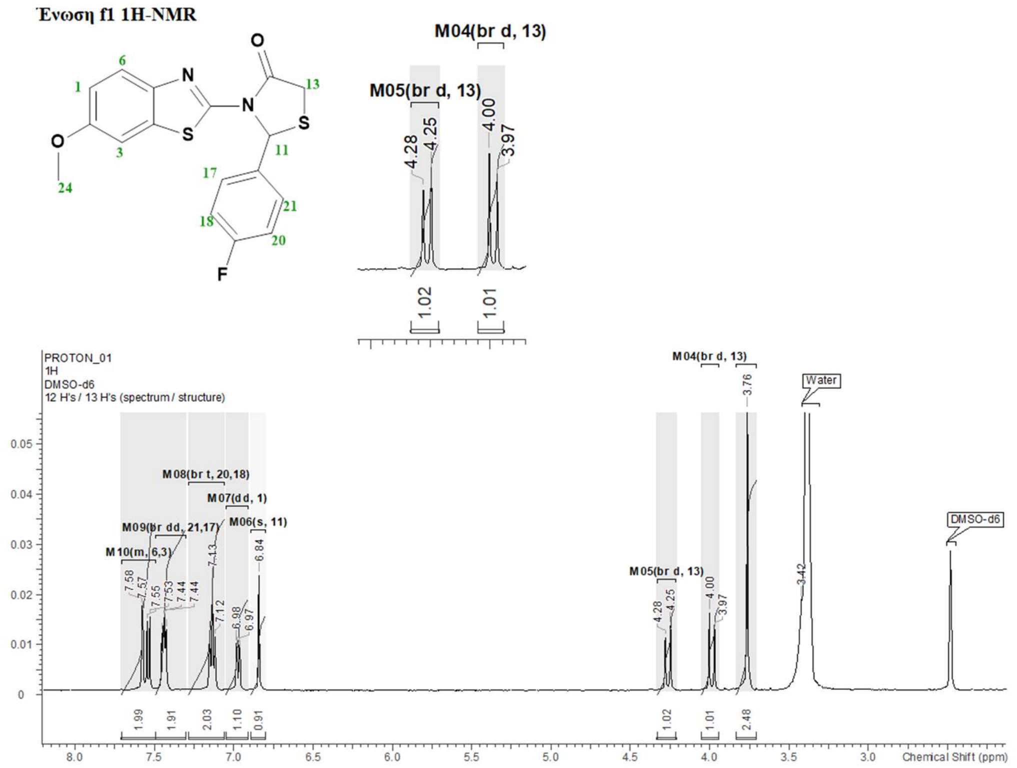
Ένωση f1 1H-NMR
M04(br d, 13)
M05(br d, 13)
PROTON_01
1H
DMSO-d6
12 H's / 13 H's (spectrum / structure)
M10(m, 6,3)
M09(br dd, 21,17)
M08(br t, 20,18)
M07(dd, 1)
M06(s, 11)
Water
DMSO-d6
Chemical Shift (ppm)

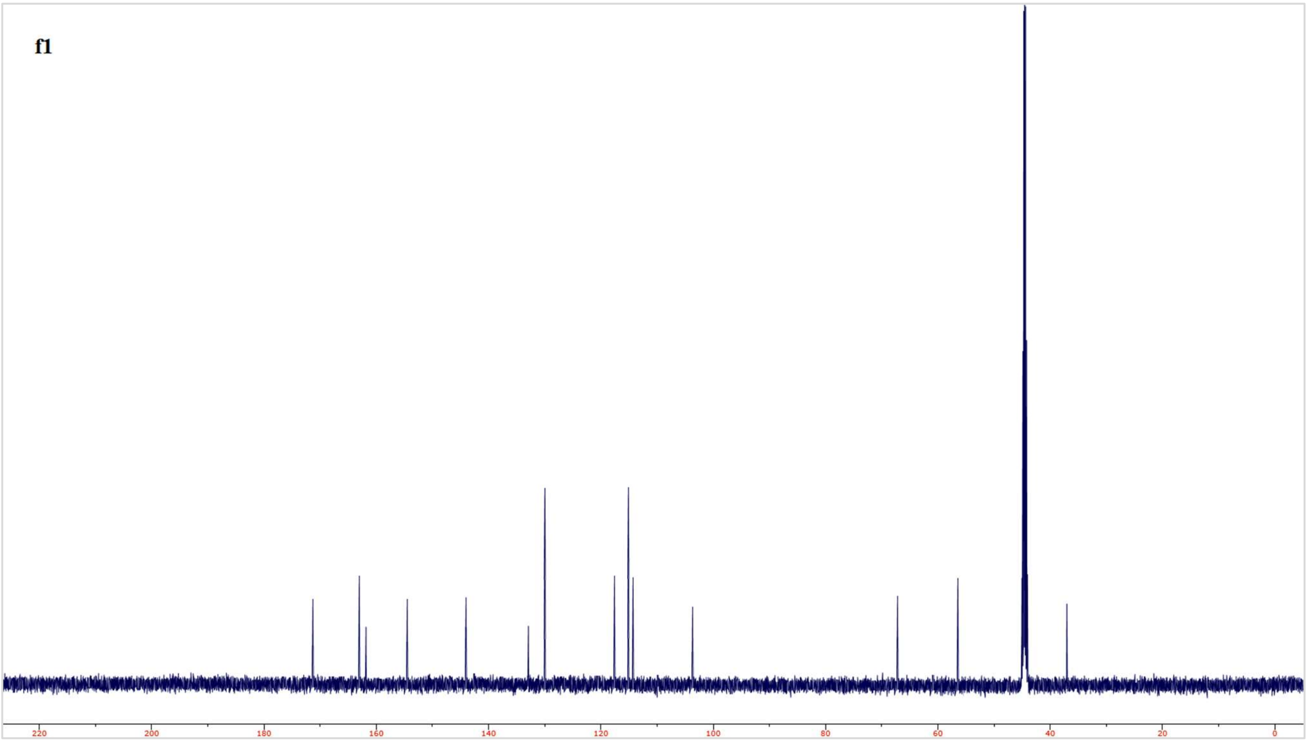
f1

Compound 8

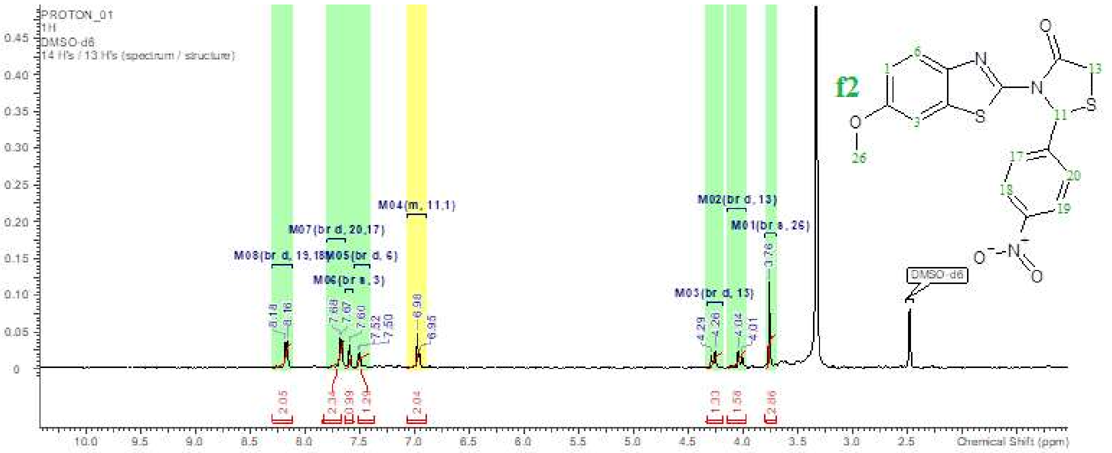
PROTON_01
1H
DMSO-d6
14 H's / 13 H's (spectrum / structure)
M08(br d, 19,18)
M07(br d, 20,17)
M05(br d, 6)
M06(br s, 3)
M04(m, 11,1)
M02(br d, 13)
M01(br s, 26)
M03(br d, 13)
DMSO-d6
f2
Chemical Shift (ppm)

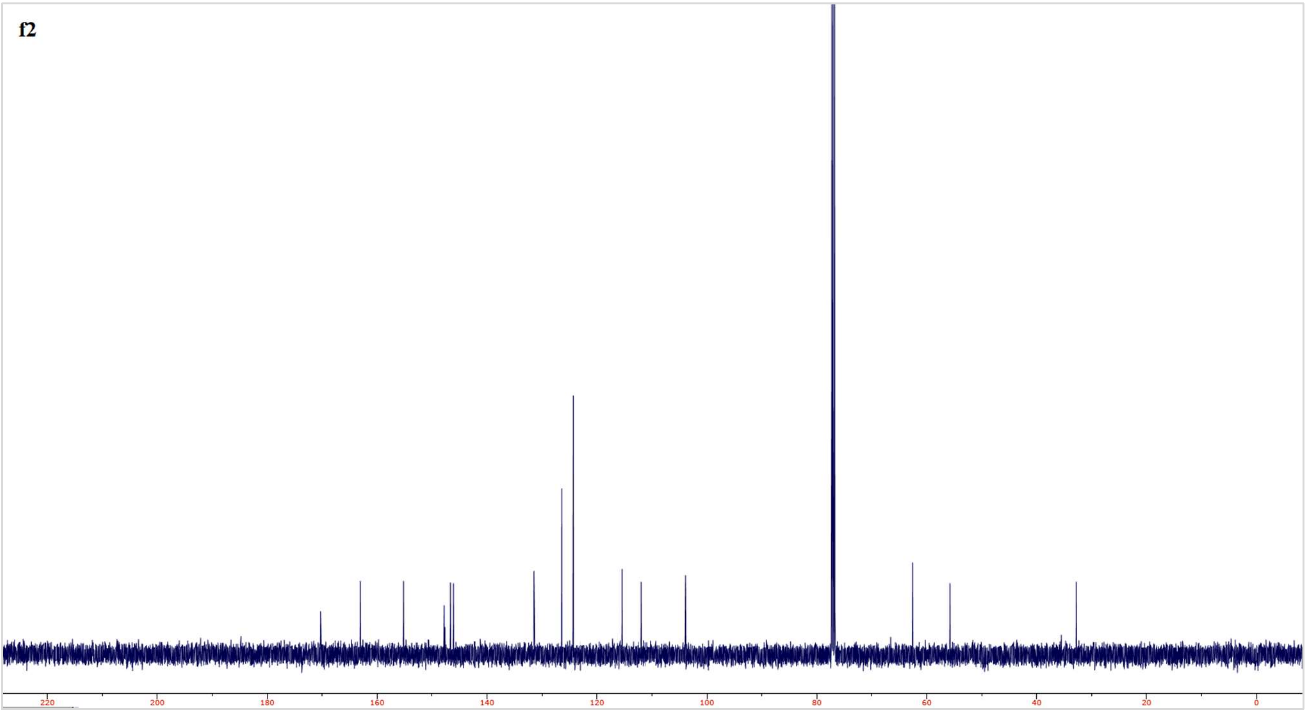
f2

Compound 9

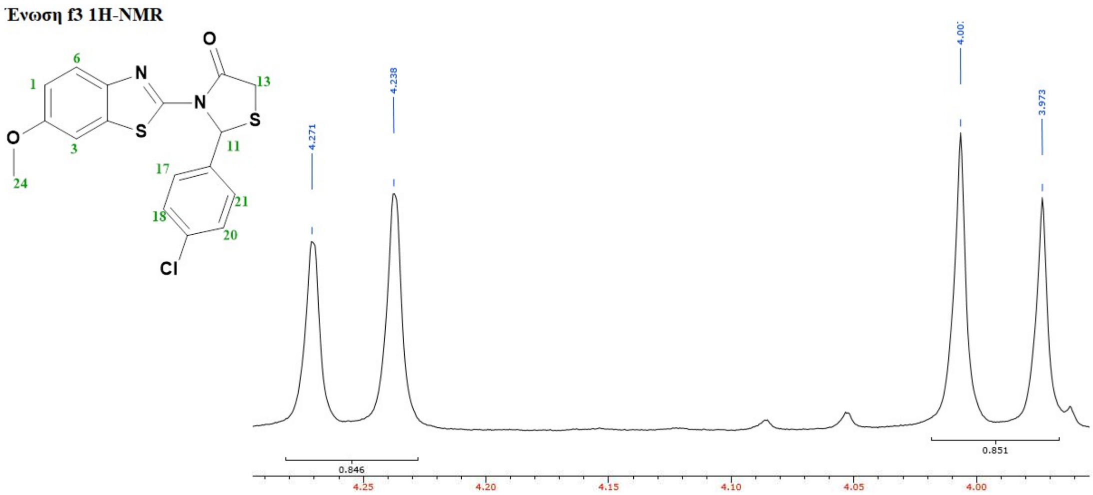
Ένωση f3 1H-NMR
4.271
4.238
4.003
3.973
0.846
0.851
4.25
4.20
4.15
4.10
4.05
4.00

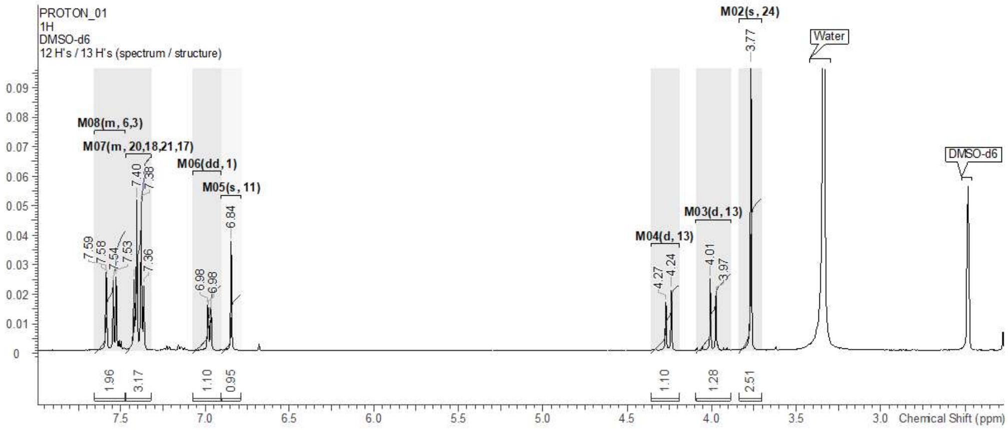
PROTON_01
1H
DMSO-d6
12 H's / 13 H's (spectrum / structure)
M08(m, 6,3)
M07(m, 20,18,21,17)
M06(dd, 1)
M05(s, 11)
M04(d, 13)
M03(d, 13)
M02(s, 24)
Water
DMSO-d6
1.96
3.17
1.10
0.95
1.10
1.28
2.51
Chemical Shift (ppm)

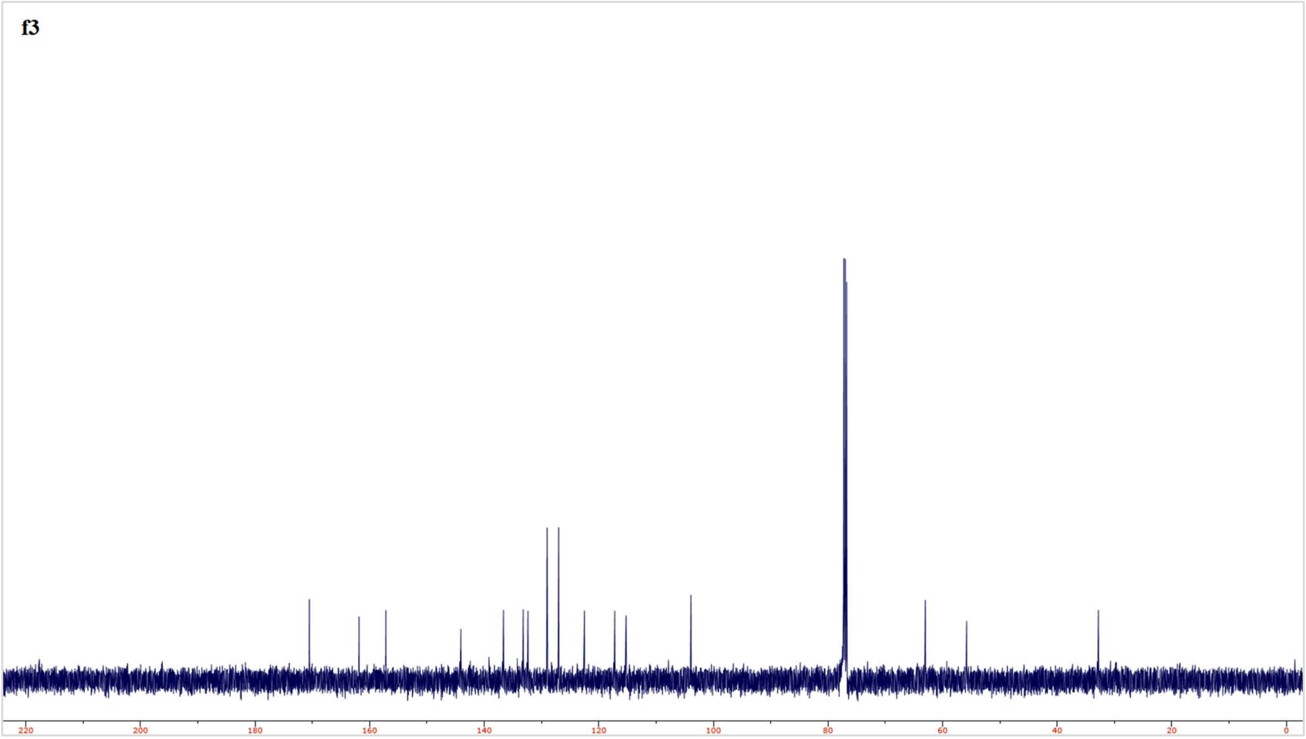
f3

Compound 10

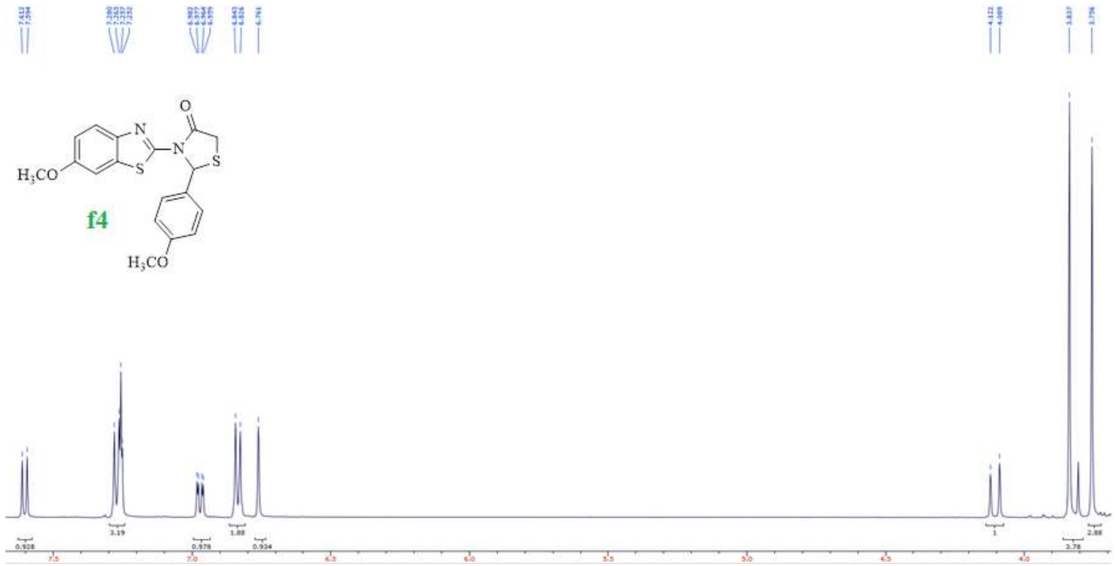
f4
H3CO
H3CO

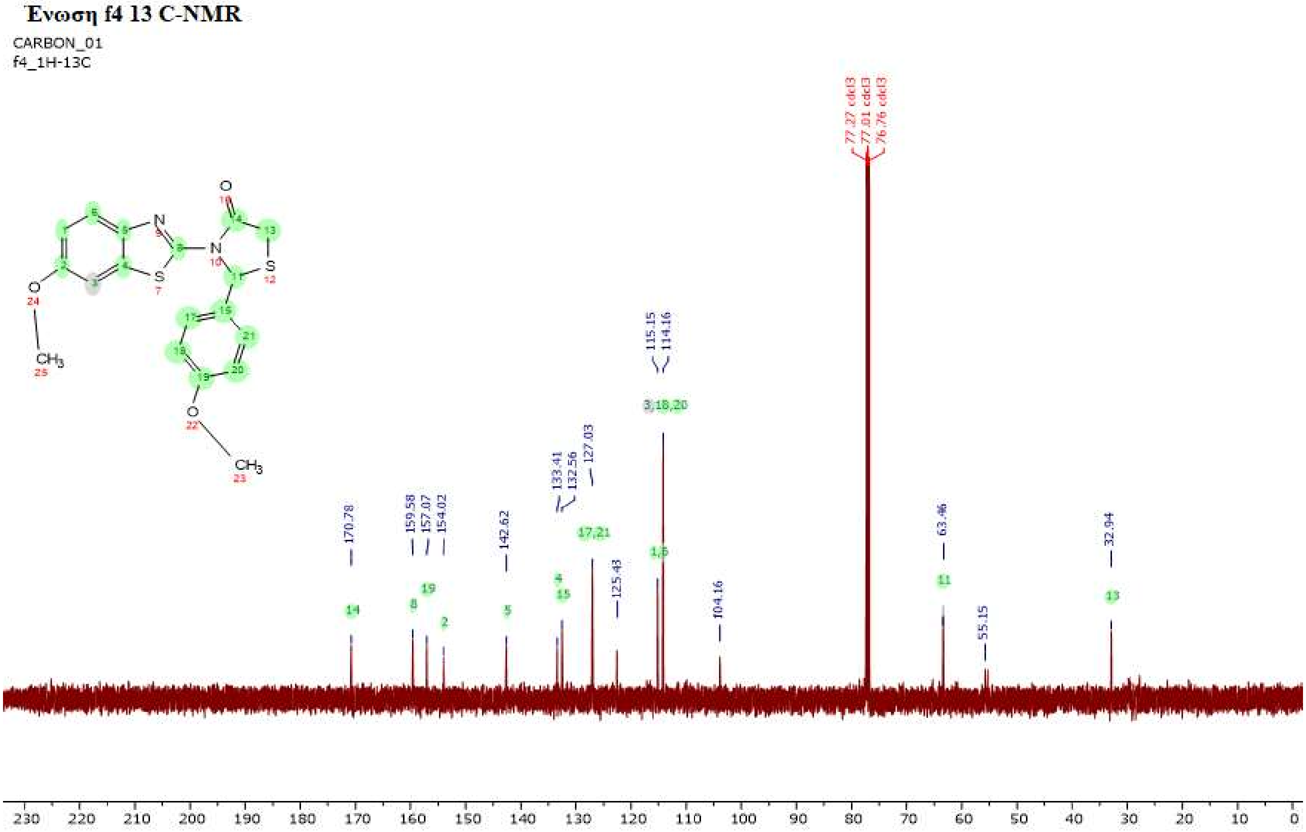
Ενωση f4 13 C-NMR
CARBON_01
f4_1H-13C
77.27 cdcl3
77.01 cdcl3
76.76 cdcl3
170.78
159.58
157.07
154.02
142.62
133.41
132.56
127.03
125.43
115.15
114.16
104.16
63.46
55.15
32.94
230
220
210
200
190
180
170
160
150
140
130
120
110
100
90
80
70
60
50
40
30
20
10
0

Compound 11

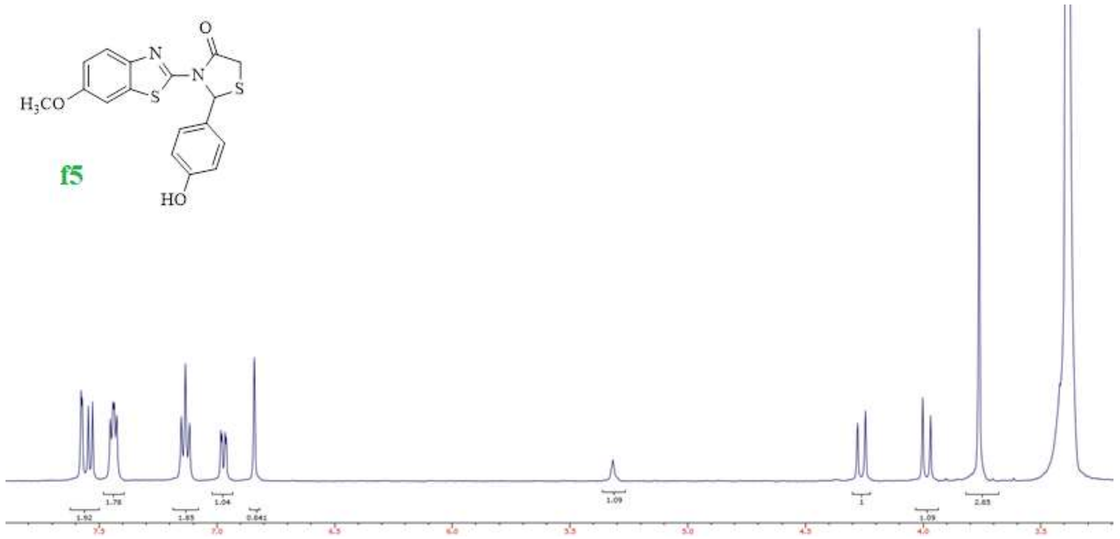

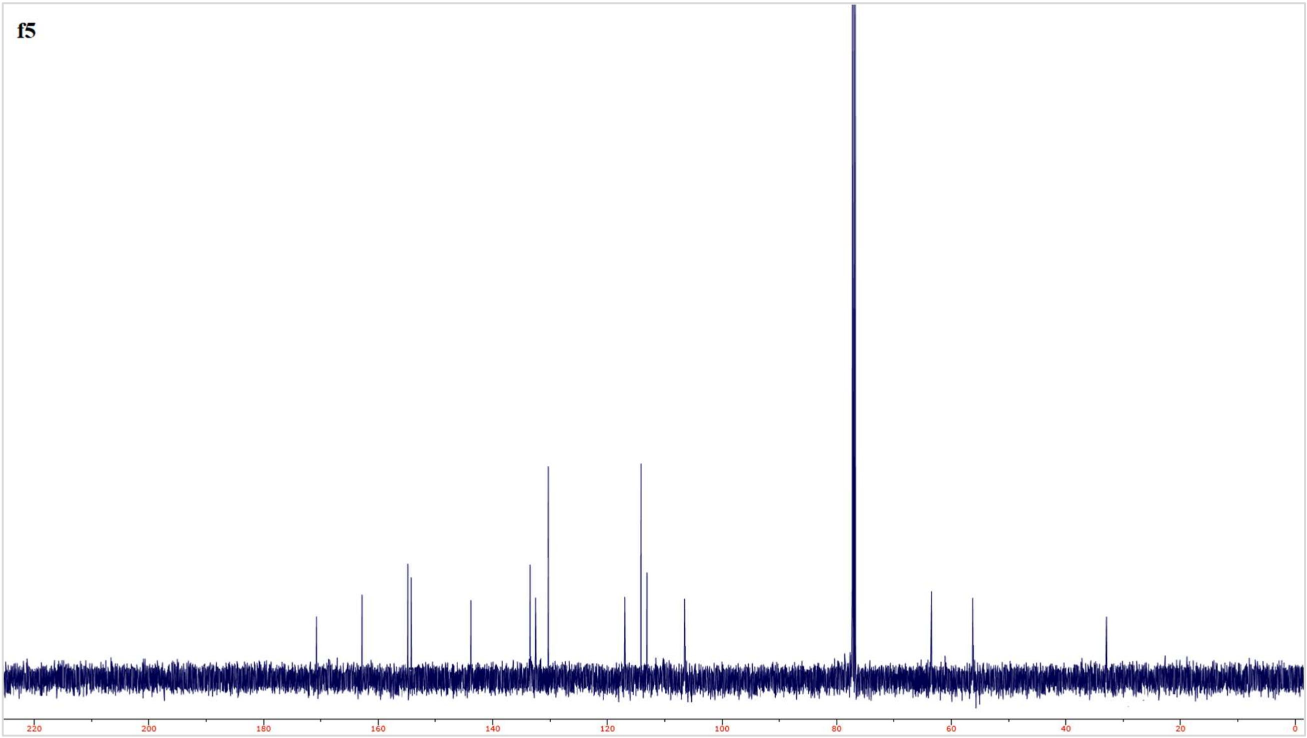

Compound 12

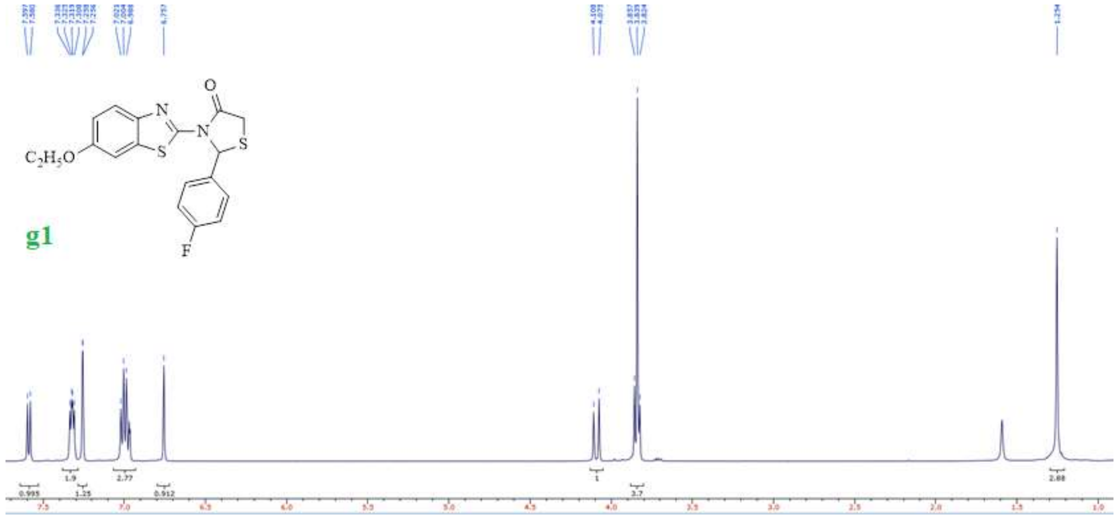

Ενωση g1 13C-NMR

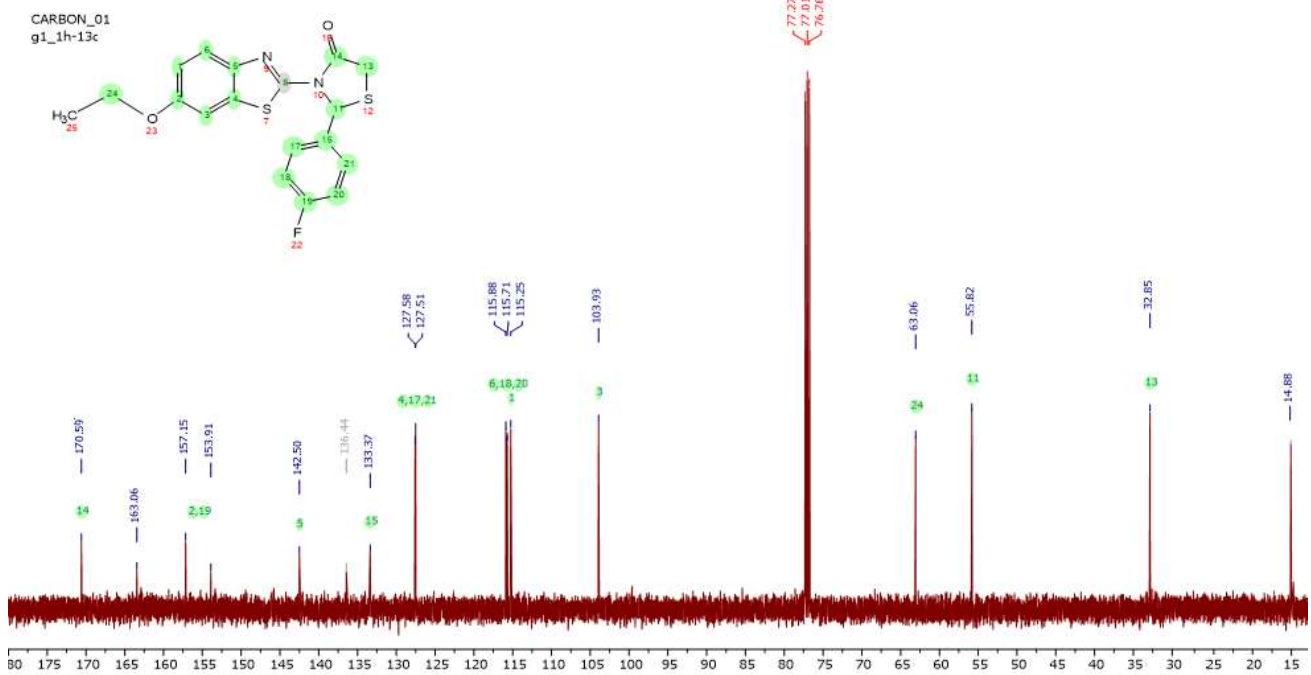

Compound 13

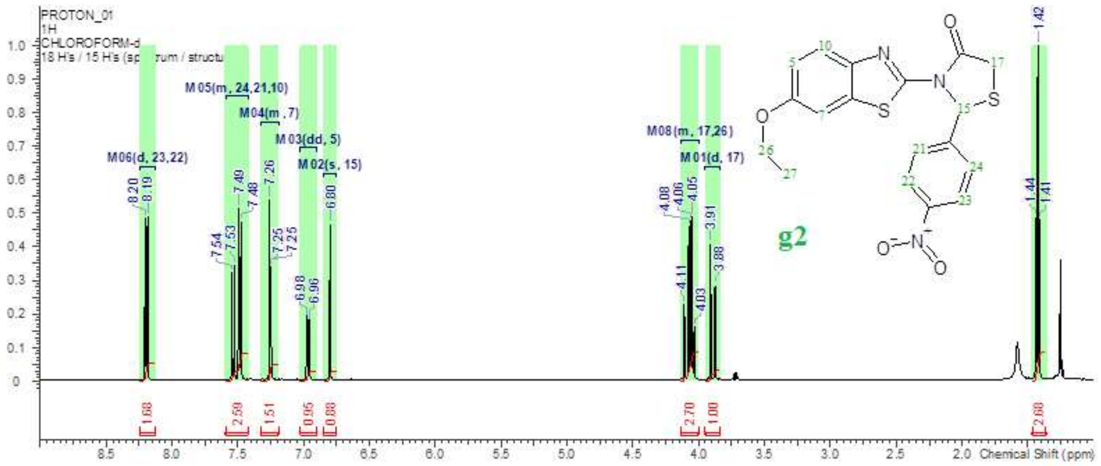

PROTON_01
1H
CHLOROFORM-d
M06(d, 23,22)
M05(m, 24,21,10)
M04(m, 7)
M03(dd, 5)
M02(s, 15)
M08(m, 17,26)
M01(d, 17)
g2
Chemical Shift (ppm)

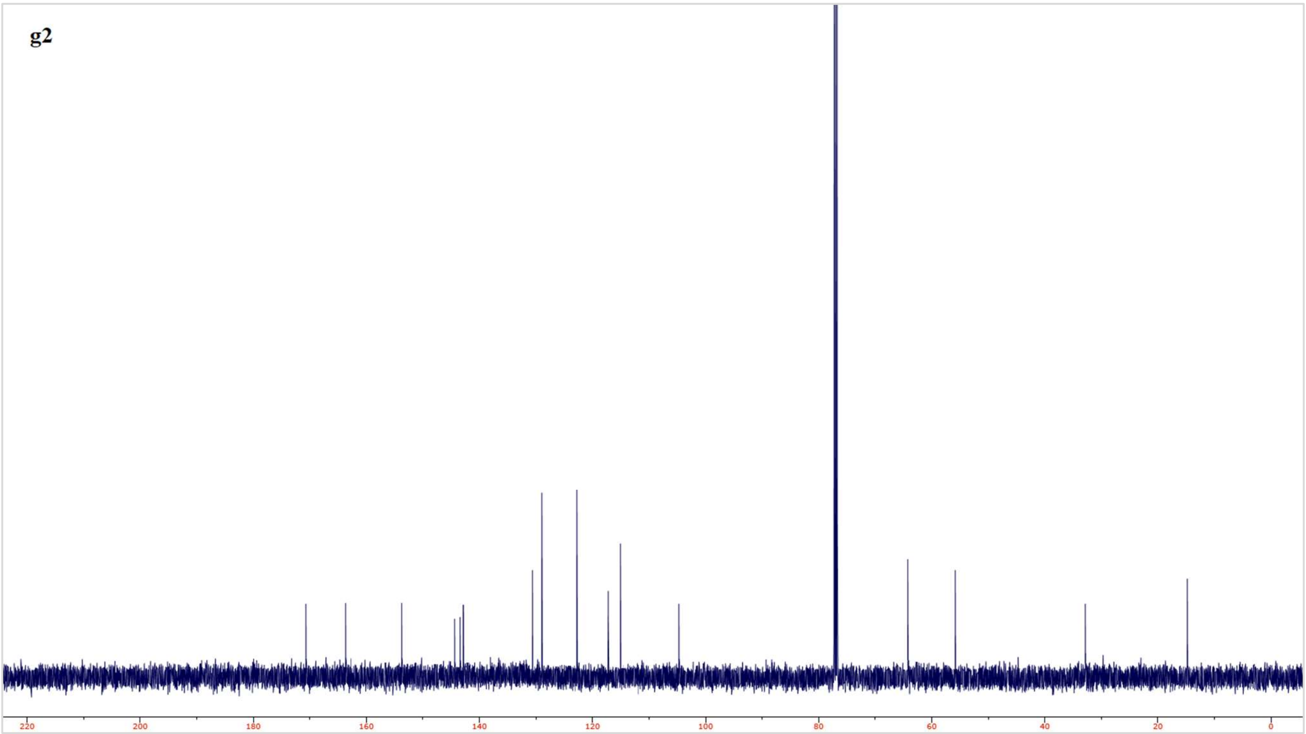

g2

Compound 14

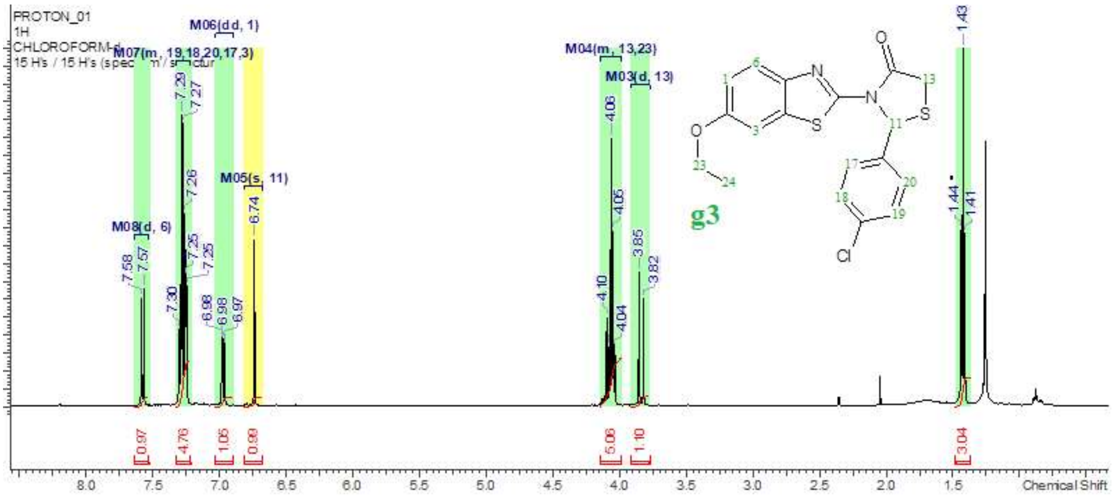
PROTON_01
1H
CHLOROFORM-d
15 H's / 15 H's (spectrum / structure)
M08(d, 6)
M07(m, 19,18,20,17,3)
M06(dd, 1)
M05(s, 11)
M04(m, 13,23)
M03(d, 13)
g3
Cl
Chemical Shift

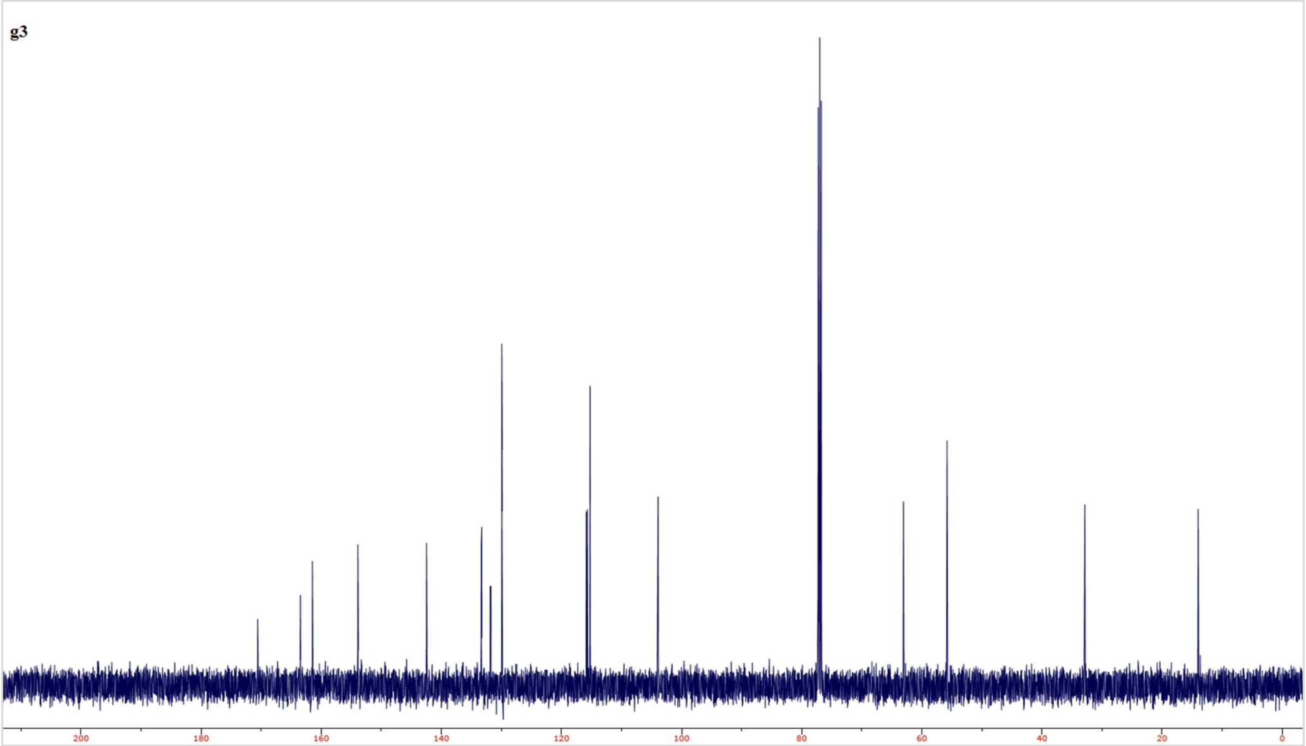
g3

Compound 15

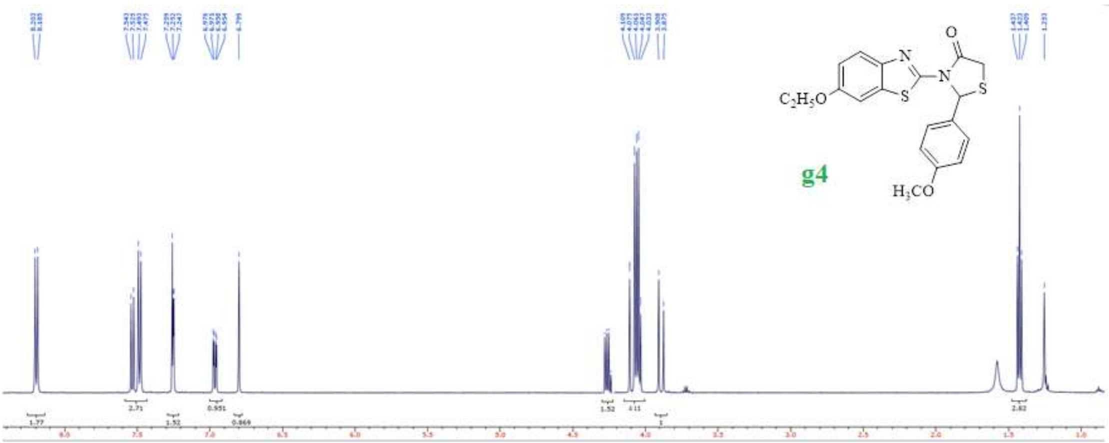

C2H5O
N
S
N
O
S
H3CO
g4
1.77
2.71
1.52
0.951
0.869
1.52
4.11
1
2.82

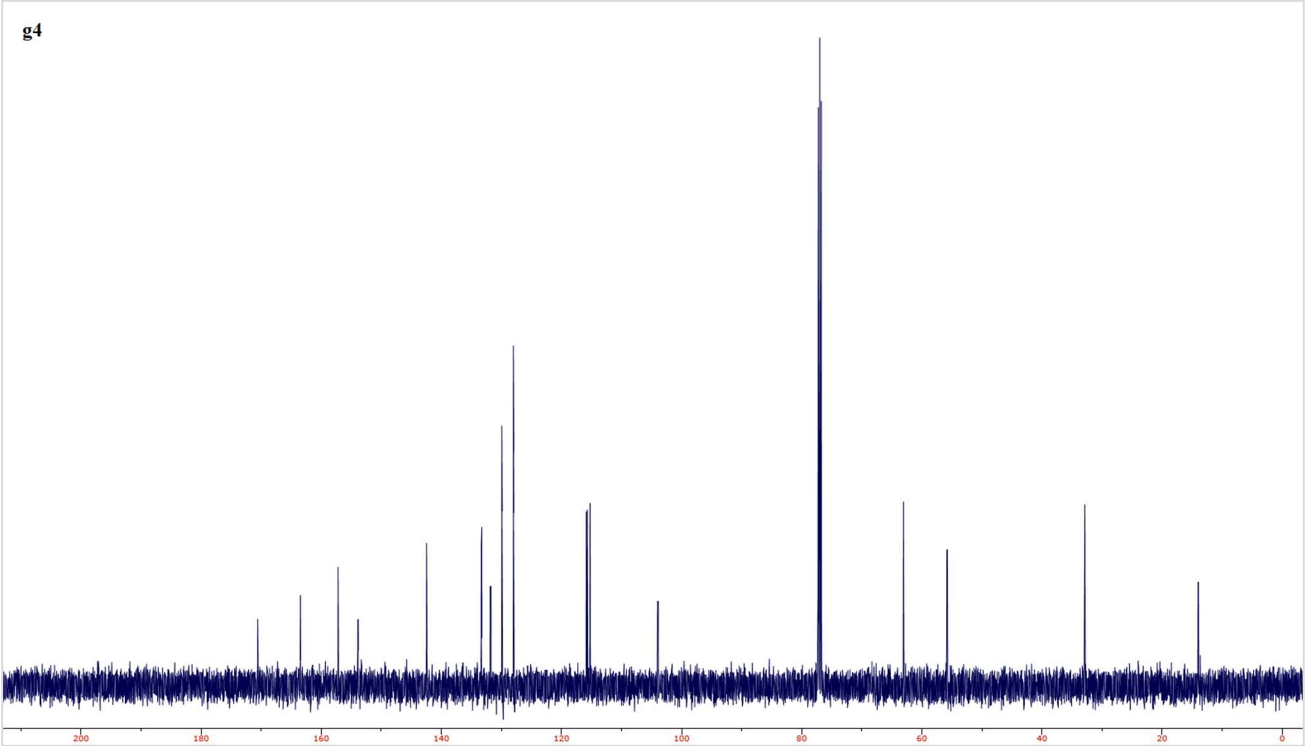

g4
200
180
160
140
120
100
80
60
40
20
0

Compound 16

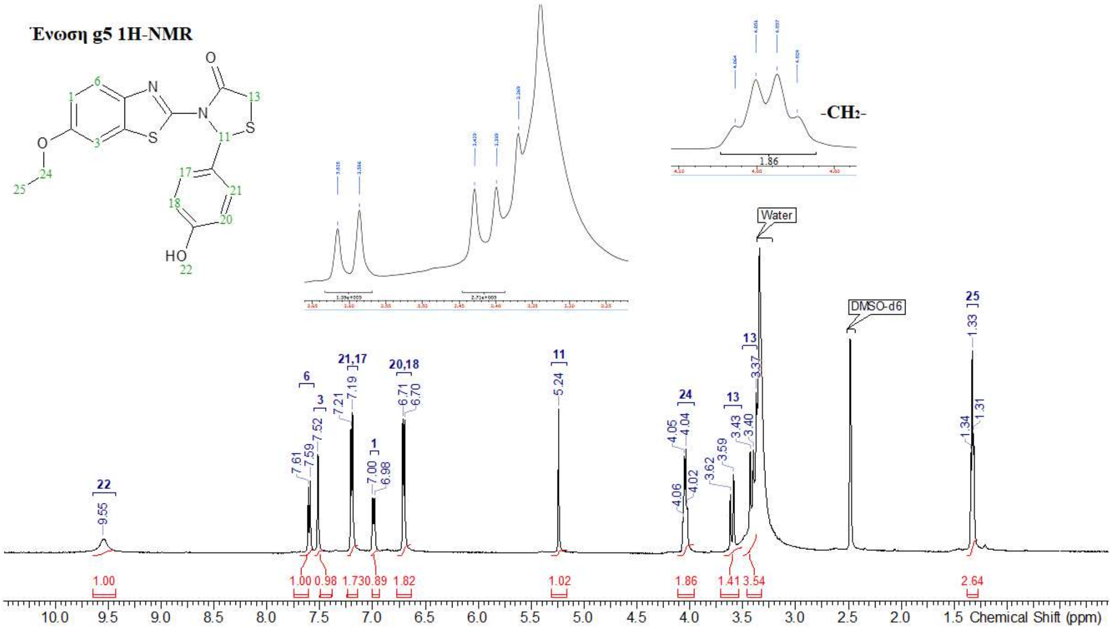

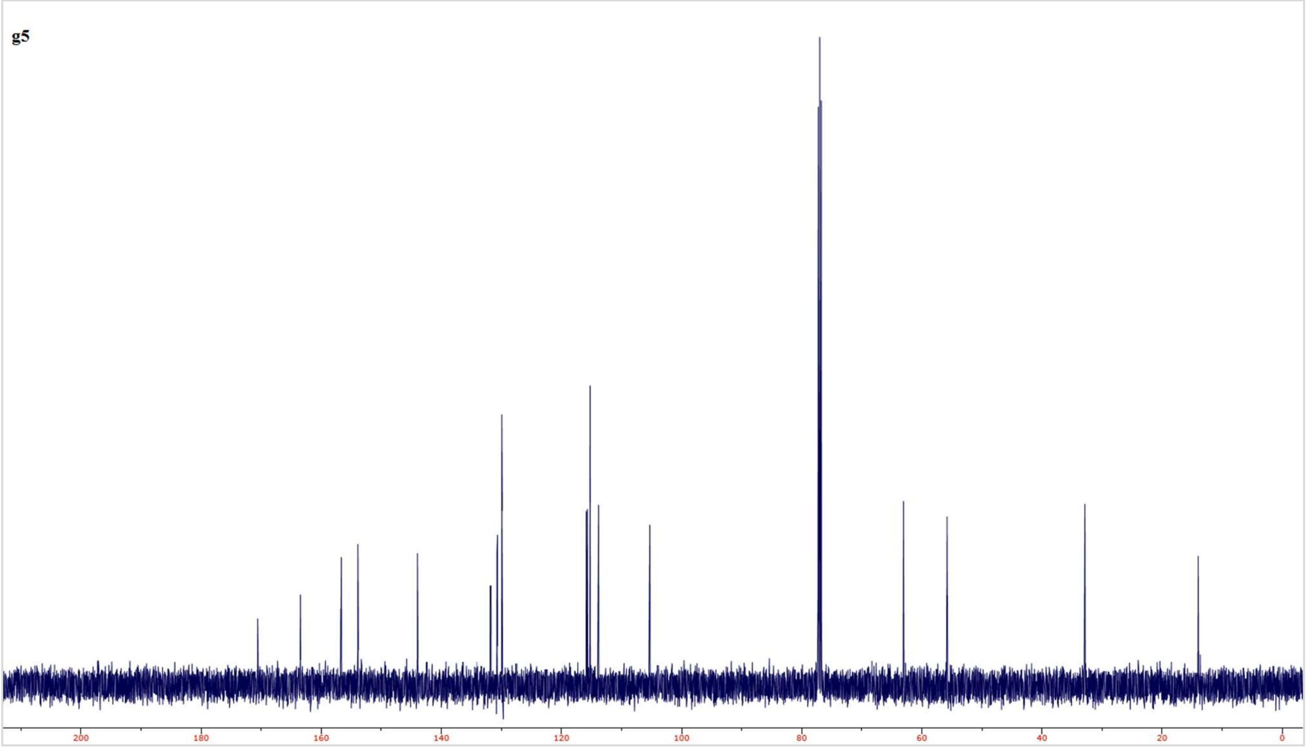

Compound 17

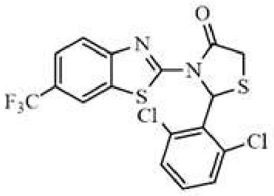

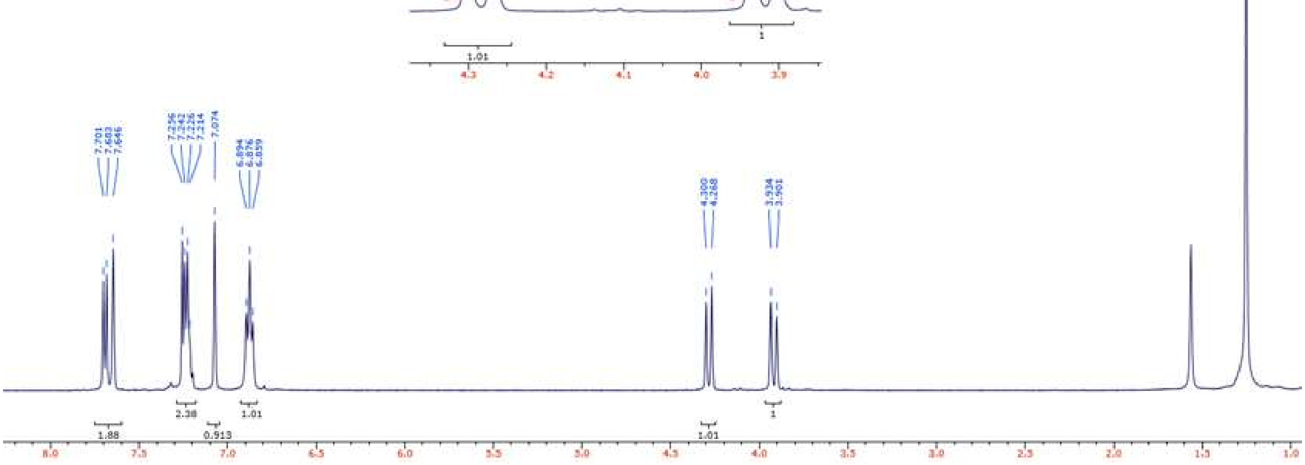

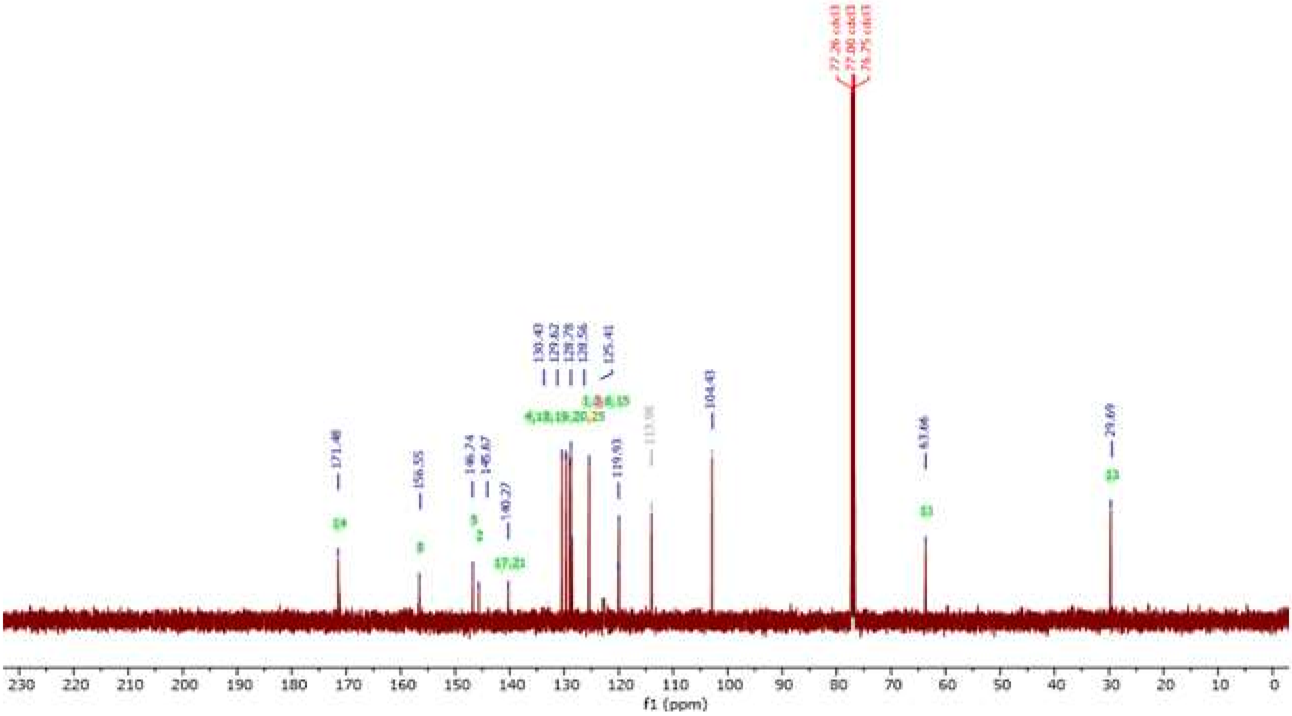

Compound 18

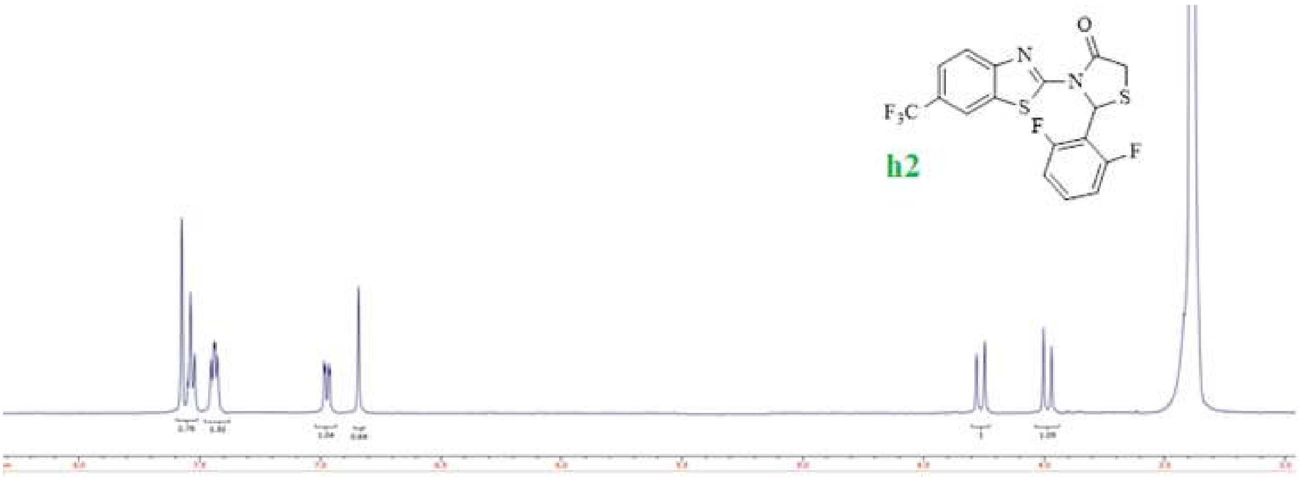

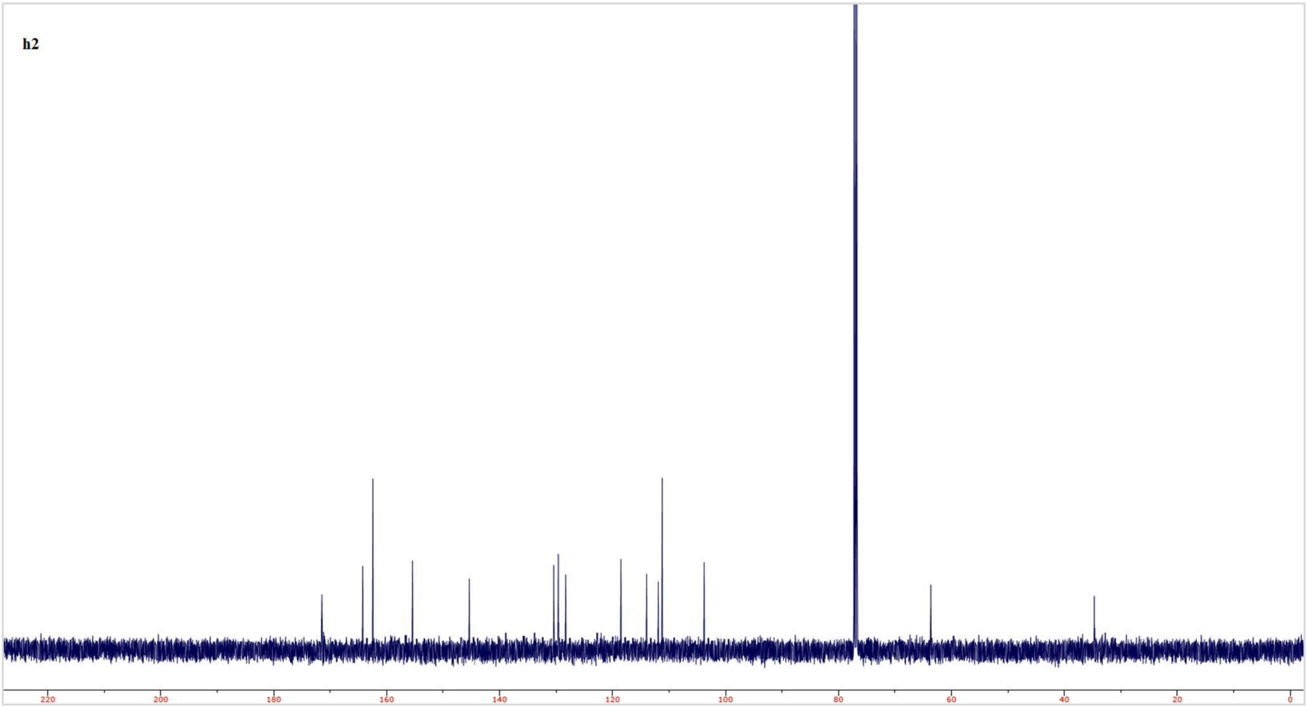

Compound 19

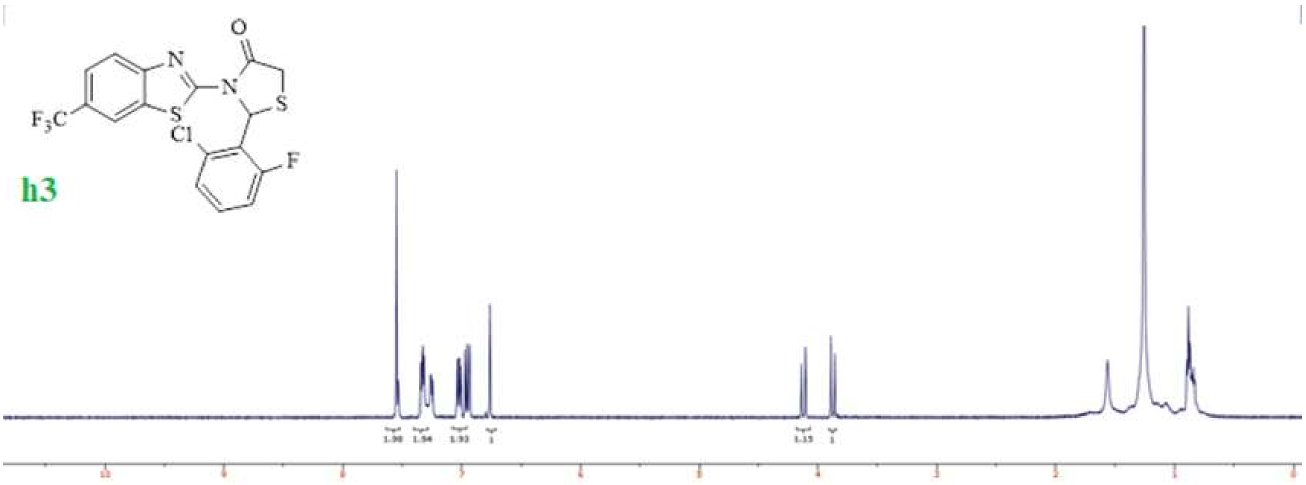

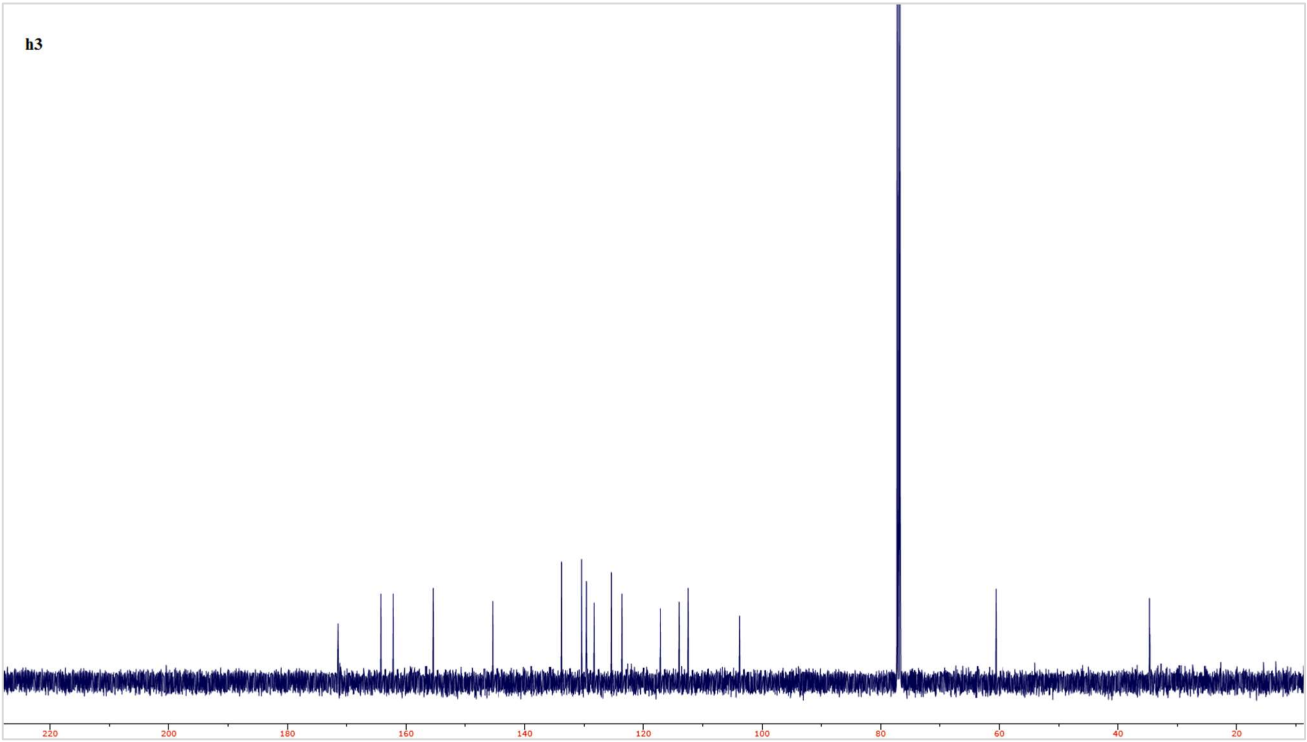

Compound 20

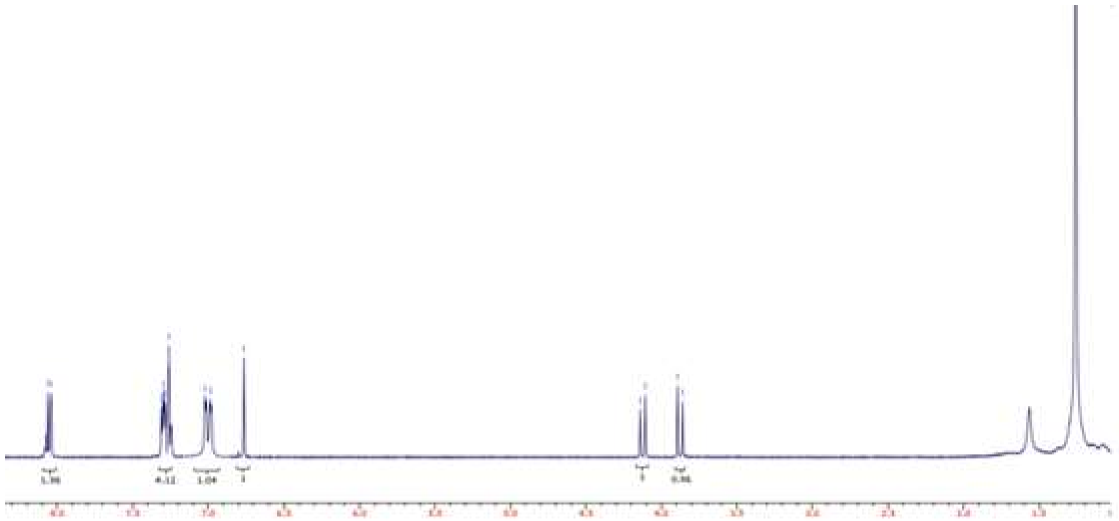

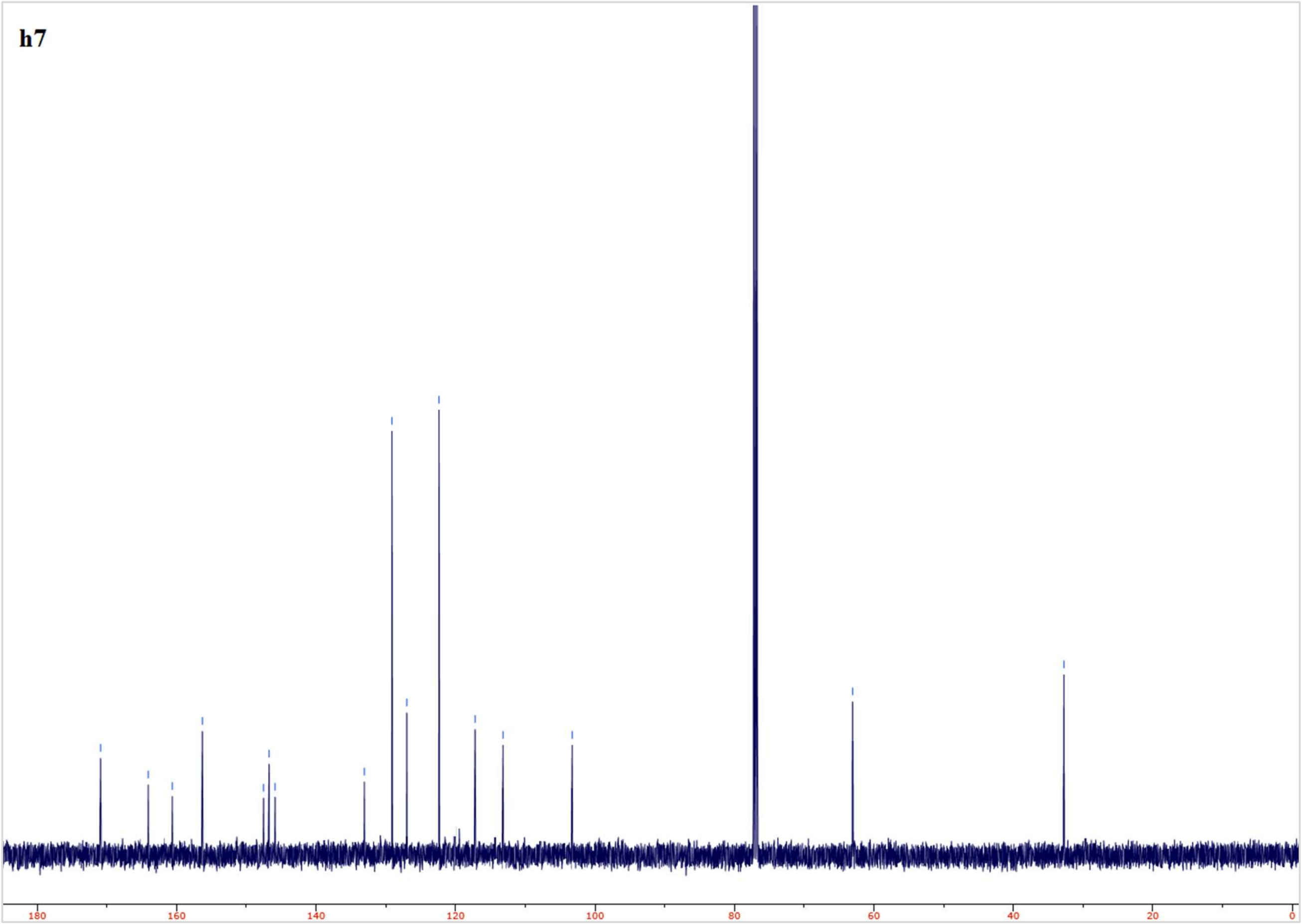

Compound 21

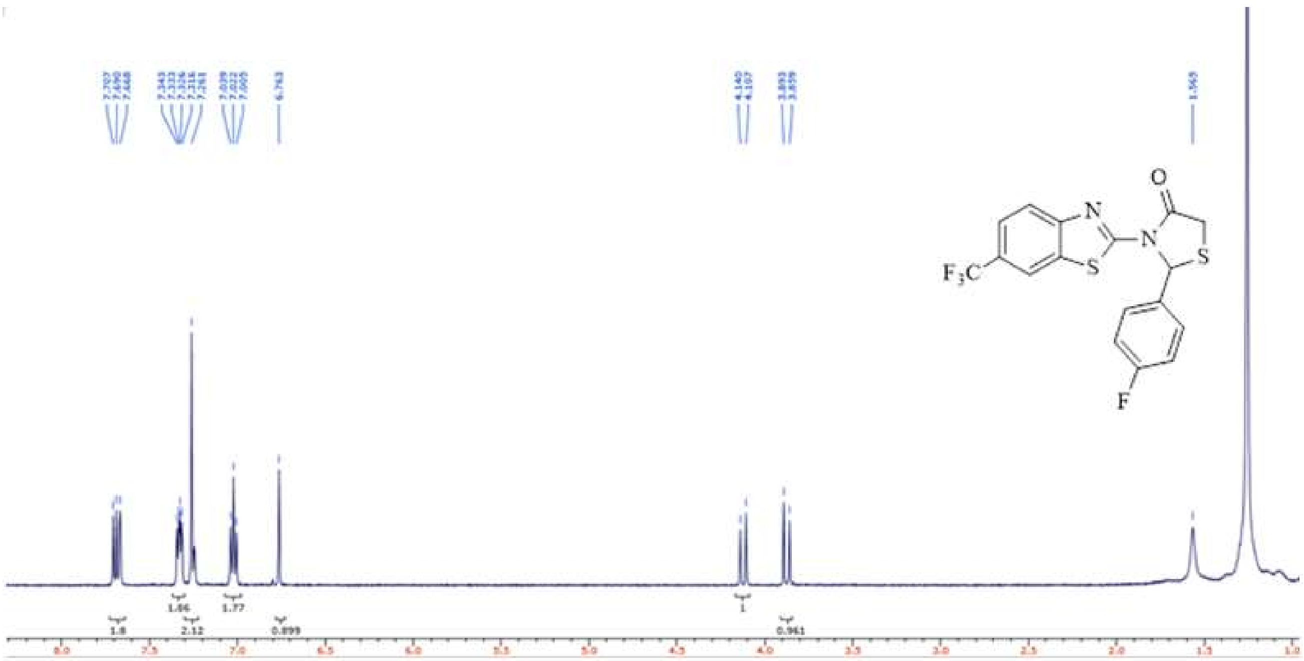

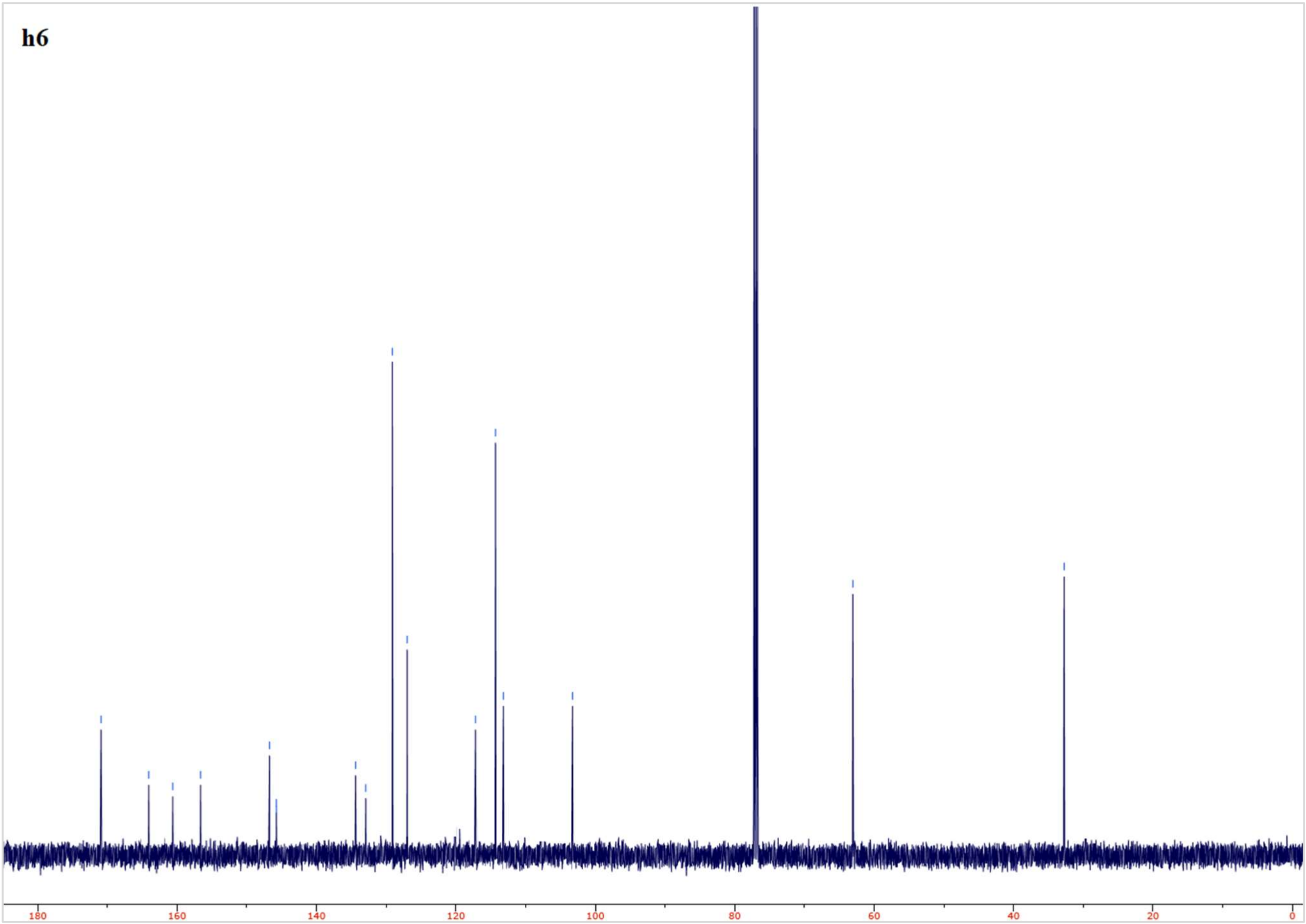

Supplement: Supplementary file 1 [file molecules-27-01930-s001.zip › molecules-1574164-supplementary.pdf]
